# A structured literature review of computer vision methods for insect identification

**DOI:** 10.1093/jisesa/ieag050

**Published:** 2026-07-30

**Authors:** Sudha Cheerkoot-Jalim, Camille Simon-Chane, Zarine Cadersaib, Zahra Mungloo-Dilmohamud, Aymeric Histace, Kavi Kumar Khedo, Leckraj Nagowah, Denis Sereno

**Affiliations:** Faculty of Information, Communication and Digital Technologies, University of Mauritius, Réduit, Mauritius; ETIS UMR 8051, CY Cergy Paris University, ENSEA, CNRS, Cergy, France; Faculty of Information, Communication and Digital Technologies, University of Mauritius, Réduit, Mauritius; Faculty of Information, Communication and Digital Technologies, University of Mauritius, Réduit, Mauritius; ETIS UMR 8051, CY Cergy Paris University, ENSEA, CNRS, Cergy, France; Faculty of Information, Communication and Digital Technologies, University of Mauritius, Réduit, Mauritius; Faculty of Information, Communication and Digital Technologies, University of Mauritius, Réduit, Mauritius; InterTryp IRD-CIRAD, Université de Montpellier, Montpellier, France; GoInsEct: Global Entomology and Infectiology Research Group, Montpellier, France

**Keywords:** AI, entomology, surveillance

## Abstract

Automated image-based identification of adult insects is increasingly critical to biodiversity monitoring, pest management, and vector surveillance, yet practical deployment remains limited by data scarcity, field variability, and fine-grained taxonomic challenges. We conducted a PRISMA-guided literature review of computer-vision methods for insect classification and identification. A Web of Science Core Collection search (31 August 2024) retrieved 930 records; after deduplication (*n *= 2) and screening, 230 articles underwent full-text quality assessment using weighted criteria for taxonomy/methods, image capture, computational technique, sample size, and performance evaluation. Of these, 111 high-quality studies met inclusion thresholds. Data were extracted on taxonomic coverage, optical devices and experimental settings, algorithms and pipelines, datasets, and outcome metrics. Deep learning dominated the field; You Only Look Once variants were common for detection and ResNet/EfficientNet/MobileNet for classification; occasional hybrids combined Convolutional Neural Network (CNN) features with traditional classifiers. CNN-based and 1-stage detectors outperformed hand-crafted pipelines; transformers and self-supervised pre-training showed promise with limited labels. Despite strong laboratory performance, generalization to field conditions was hindered by illumination, occlusion, and pose variability. Public datasets were scarce and geographically skewed, limiting reproducibility and equitable benchmarking. Taxonomic coverage concentrated on Lepidoptera, Diptera, Hemiptera, and Coleoptera. We recommend advancing the field through comprehensive reporting beyond overall accuracy, the design of lifecycle-aware and domain-adapted models validated under field conditions, the establishment of diverse benchmarks with standardized imaging protocols, and the development of interpretable architectures suitable for deployment in embedded trapping systems.

## Introduction

Insect classification is a cornerstone of ecological research, biodiversity monitoring, pest management, and disease vector surveillance. Historically, taxonomy is mainly based on adult-stage morphological characters, whereas immature stages require specialized microscopic imaging and sampling procedures because they occur in diverse environmental compartments. Computer-vision identification of immature stages, in turn, relies on different methods compared to adults. The adult stage identification relied on expert examination of morphological traits such as wing venation, body segmentation, and pigmentation under microscopes. While effective, these manual methods are labor-intensive, time-consuming, and constrained by the scarcity of taxonomic expertise (Hebert et al. 2003). DNA barcoding emerged as a molecular alternative, offering precision through genetic markers, but its high costs and logistical barriers hinder large-scale field applications (Hebert et al. 2003). These limitations have spurred interest in automated, image-based classification systems powered by advances in computer vision and deep learning (DL), which promise scalable, cost-effective solutions for biodiversity, agricultural, and public health challenges.

Recent breakthroughs in Artificial Intelligence (AI), particularly deep neural networks (DNNs), have transformed insect taxonomy. Models like ResNet and MobileNetV2 excel at analyzing morphological features in images, achieving high accuracy in distinguishing families such as Calliphoridae (Ong and Hamid 2022). Innovations like self-supervised learning (SSL), where models learn from unlabeled data to reduce annotation demands, and hybrid architectures integrating transformers (attention-based models adept at capturing long-range patterns) further enhance robustness (Grill et al. 2020). For instance, techniques such as Bootstrap Your Own Latent have proven effective in low-data regimes, a common hurdle in entomology (Grill et al. 2020, Charoenpanyakul et al. 2024). Beyond visible-spectrum imaging, hyperspectral sensors, which capture hundreds of narrow spectral bands to analyze chemical and material properties, are being used to detect subtle spectral signatures in insect exoskeletons (Tan et al. 2024). Separately, Wing Interference Patterns (WIPs), microstructural features that generate iridescent colors through light interference, provide taxonomically diagnostic structural traits (Cannet et al. 2024). Despite these advancements, critical challenges persist. First, the scarcity of large, well-annotated datasets limits model generalizability, particularly for rare species. Second, environmental variability, such as lighting, occlusion, and orientation in field images, degrades classification accuracy. Third, fine-grained species discrimination remains problematic for taxa with subtle morphological differences, such as cryptic mosquito species. Finally, deployment of DL models in resource-constrained settings faces hurdles like high computational costs and “black-box” decision-making, which complicates trust in automated systems.

To address these challenges, researchers are pursuing multifaceted strategies. SSL frameworks like momentum contrast minimize reliance on labeled data by pre-training on uncurated field images (He et al. 2020), while citizen science initiatives like iNaturalist crowdsource annotated datasets (Silvertown 2009). Meanwhile, hyperspectral imaging enhances classification by capturing species-specific spectral fingerprints, while WIP analysis leverages nanoscale wing structures to distinguish morphologically similar species (Cannet et al. 2024, Tan et al. 2024). However, prior reviews have not systematically evaluated how these emerging techniques collectively address field deployment barriers or bridge the gap between computational innovation and practical entomological needs.

This review evaluates optical devices, computational pipelines, datasets, and performance factors across 111 high-quality studies to map methodological trends, identify gaps, and outline future directions for scalable computer vision-based adult insect classification.

Four research questions (RQs) have been set up for this paper. The first, **RQ1**, “How broad is the taxonomic and biodiversity coverage in current image-based insect identification research?,” addresses the taxonomic and biodiversity coverage. The different imaging systems and data acquisition methods are covered through **RQ2**, “What imaging systems and data acquisition methods are used for insect image capture?.” **RQ3**, “Which insect image datasets are available, and what are their characteristics (size, accessibility, geographic coverage)?,” focuses on the datasets for this domain. Finally, **RQ4**, “What computer vision and image processing techniques have been applied for insect classification?,” centers on computer vision algorithms.

## Materials and Methods

This structured literature review was informed by PRISMA (Preferred Reporting Items for Systematic Reviews and Meta-Analyses) guidelines for literature search and reporting transparency (Nakagawa et al. 2017, Page et al. 2021).

### Search Strategy

#### Information Sources

The information source considered was the Web of Science (WoS) Core Collection. WoS indexes high-impact, peer-reviewed journals and conference proceedings, thus ensuring the reliability and credibility of sources. The reference management tool used for this study was SciWheel (later renamed LeanLibrary Workspace), which, through its web application, allows easy import of citations and sharing of references and notes among collaborators.

#### Search

The search query was: “(insect OR insecta) AND (classification OR identification) AND (‘image processing’ OR ‘computer vision’ OR ‘AI’ OR ‘deep learning’) (All Fields).”

This query was designed to capture all studies relevant to automated insect recognition. The inclusion of “insect” (common term) and “Insecta” (scientific name) ensured comprehensive coverage of the taxonomic group of interest. The terms “identification” and “classification” were selected to encompass studies addressing either taxonomic labeling or automated recognition tasks. Finally, the computing-related terms “image processing,” “computer vision,” “AI,” and “deep learning” were included to target the main technological approaches applied to image-based classification. The search was applied to all fields to avoid excluding relevant studies in which these keywords might not appear in the title but are discussed elsewhere in the record.

The systematic database search was conducted on 31 August 2024 without applying any restriction on publication date to ensure completeness and accuracy of the results. One author performed the search in the WoS Core Collection using the predefined search terms. The retrieved citations were exported in BibTeX format and subsequently imported into SciWheel for organization and screening.

### Eligibility Criteria and Study Selection

The study selection strategy was carefully designed to capture all relevant research while minimizing the risk of selection bias.

#### Inclusion and Exclusion Criteria

A set of pre-defined inclusion and exclusion criteria was applied to systematically identify eligible studies.

Inclusion criteria:

- Peer-reviewed articles focusing on the application of image processing or computer vision techniques for the identification or classification of insects.
- Studies describing the use of relevant AI techniques for insect identification or classification using images.
- Studies that were carried out based on insects’ images.
- Articles like primary studies.

Exclusion criteria:

- Articles not focusing primarily on insect classification.
- Studies carried out only on images of eggs or larvae of insects.
- Studies that performed classification based on acoustic data.
- Opinions, letters, and comments, as well as secondary studies, like reviews and meta-analyses.

### Article Screening

After removing duplicates, the remaining articles underwent a structured screening process. Six authors participated in the screening of articles from the SciWheel platform. Articles were equally distributed among the authors, who independently reviewed the title and abstract of each assigned article. The decision to retain an article was based on the inclusion and exclusion criteria listed above. To minimize bias and ensure consistency in the selection process, each author (A1) reviewed the work of another author (A2) by re-assessing 20% of the articles assigned to A2. Any discrepancies were resolved through group discussion during consensus meetings involving all authors. The set of pre-selected articles was then subjected to a subsequent quality assessment.

### Quality Assessment

The quality assessment phase was conducted to ensure that the results and recommendations of the structured literature review were grounded in rigorous and reliable research. Seven authors were each assigned an equal number of pre-selected articles, which they evaluated in full for quality. Each article was given a score based on a set of predefined quality criteria (QC): (i) QC1—taxonomy/methodology, (ii) QC2—image capture approach, (iii) QC3—image processing or computer vision technique, (iv) QC4—number of processed samples, and (v) QC5—performance evaluation. A weighting factor of 2 was assigned to QC3 and QC5, as these criteria represent the primary focus of this study, while the remaining 3 criteria were assigned a weighting of 1. The detailed quality assessment criteria and the scoring scheme applied can be found in Supplementary Material S1.

The quality assessment revealed that most articles achieved high scores for QC3, QC4, and QC5. However, greater variability was observed in QC1 and QC2. Several studies adopted a “computer vision–only” approach, in which taxonomy and image capture aspects were not addressed, and the focus was primarily on achieving high recognition accuracy, often independent of application relevance. To exclude such “application-agnostic” studies, only articles that scored at least 1 in both QC1 and QC2 were retained for further analysis.

Based on the assigned scores, each article was classified as either high quality (total score: 11 to 14) or low quality (total score: <11). Only high-quality articles were retained for in-depth analysis. The quality assessment process thus yielded the final set of articles included in the structured review.

### Data Extraction

We used Elicit (Whitfield and Hofmann 2023), an AI-powered literature review tool developed by Ought, to assist in data extraction from scientific publications. Elicit was employed to collect structured information across 6 key dimensions: Methodology, Outcome measurement, Dataset description, Algorithms, Accuracy, and Optical devices used. The extraction process was carried out on the curated set of peer-reviewed articles relevant to our research focus.

To ensure the reliability of the extracted data, all outputs were manually reviewed by at least 2 researchers. Discrepancies were resolved through consensus discussions. This semi-automated approach facilitated a consistent and reproducible synthesis of technical information critical to our comparative analysis.

The data extracted and curated were then grounded into a data collection sheet.

### Geographic Distribution Visualization

To visualize the geographical distribution of published studies, we generated a static world map displaying the number of articles per country. Data were manually compiled based on the structured literature review, with each article associated to 1 or more countries according to the location where the study was performed or the institutional affiliation of the authors. A country-level count of articles was created, and the resulting dataset was processed using Python. The map was generated using the Plotly Express (https://plotly.com/python/plotly-express/) library, which enables the creation of customized choropleth maps. Each country with at least 1 study was highlighted, and the corresponding number of studies was displayed directly on the map. The final map was exported as an image file (JPEG) for inclusion in the manuscript. This visualization provided a concise overview of the global distribution of research activity included in our analysis.

## Results and Discussion

### Selection Process Overview

**Fig. 1.**
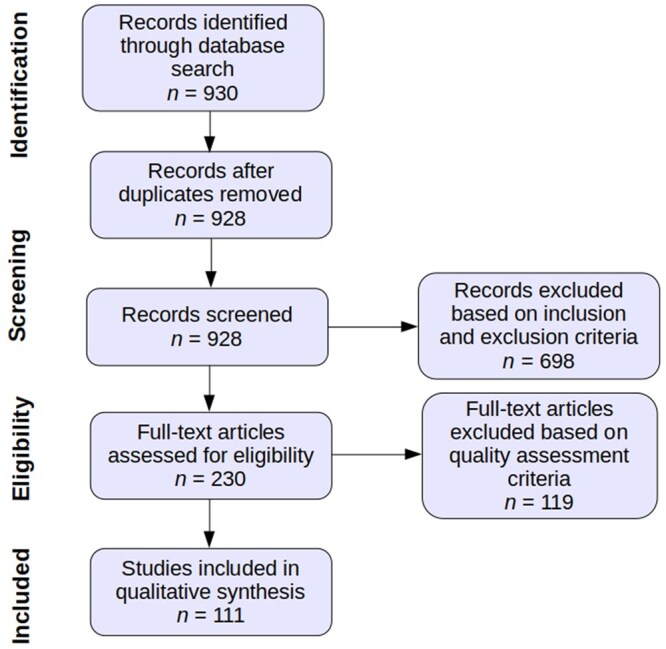
PRISMA flowchart.

The search on WoS yielded a total of 930 records. After removing duplicates (2), 928 studies were considered for the article-screening process. Abstracts were then screened based on the inclusion and exclusion criteria, resulting in 230 articles for further analysis. A quality assessment was subsequently conducted on the full texts of these articles. The final review was based on 111 articles, representing approximately 12% of the original records identified. Figure 1 depicts the PRISMA flowchart outlining the identification, screening, and inclusion processes followed in this structured literature review.

The data collection sheet, which includes the list of studies retained for the final review along with the extracted and curated data, can be found in Supplementary Material S2. The data fields were defined according to the objectives of the review and represent the key information required to address the RQs formulated for this study.

### Timeline of Published Papers

**Fig. 2.**
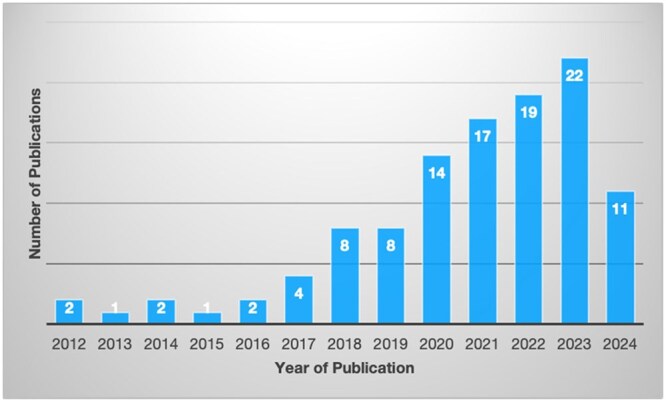
Annual distribution of publications on automated insect identification (2012 to 2024).

The timeline of publications reveals a clear upward trend in research activity over the past decade (Fig. 2). From just a handful of papers between 2012 and 2017, the field began to gain momentum starting in 2018. This growth accelerated steadily after 2019, with a sharp rise in output from 2020 onward. The most active years were 2021 to 2023, with 2023 seeing over 22 publications. As of August 2024, a total of 11 papers had already been published, indicating sustained interest. This surge reflects both recent technological advancements in AI and computer vision, as well as the growing recognition of automated monitoring as a crucial tool for biodiversity research and agricultural pest management. Since the search strategy included the terms “AI” and “deep learning,” studies that did not primarily focus on insect classification, as well as those conducted exclusively on eggs or larvae, were excluded. These criteria could explain the relatively low number of articles retained prior to 2018.

### Geographical Coverage of the Studies

**Fig. 3.**
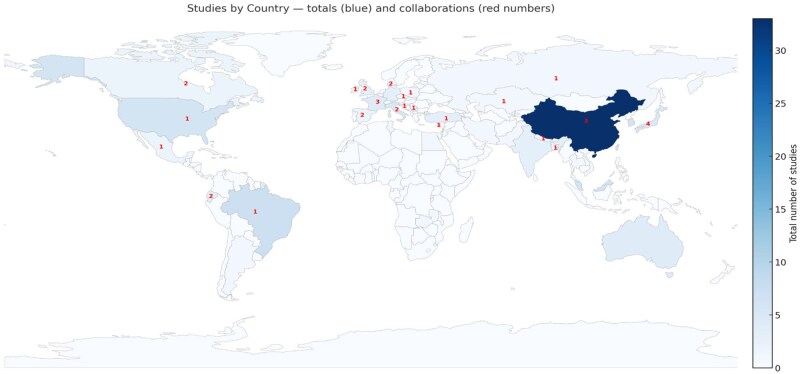
Geographic distribution of institutions carrying automated insect identification studies (*n* = 111). Blue shading: total number of studies per country. Red numbers: studies that were collaborations (ie the study listed ≥2 countries).

The most represented country, in the selected set of scientific papers, is China, particularly the provinces of Yunnan, Shandong, Guangdong, and Zhejiang. Other frequently cited countries include the United States, India, Brazil, and Australia. Multinational collaborations also appear, reflecting a globally shared interest in AI-driven entomological research (see Fig. 3).

However, a notable gap exists: many highly biodiverse regions, especially across sub-Saharan Africa, are underrepresented or absent. Despite their ecological importance and rich insect fauna, most African countries were not featured in the studies reviewed (see Fig. 3). This highlights an important geographic bias in current research efforts and underscores the need for more inclusive, globally distributed scientific inquiry into insect biodiversity.

**Fig. 4.**
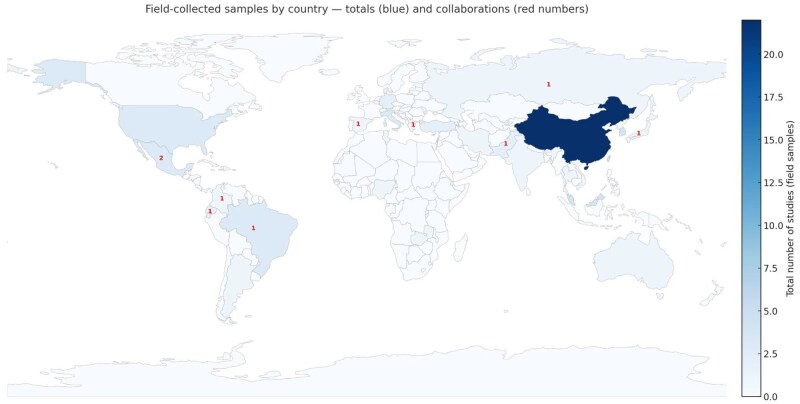
Geographic origin of samples processed for automated insect identification. Only studies that use specific samples were processed. Blue shading: total number of studies with field samples. Red numbers: studies where field samples were collected with at least 1 other country involved.

Similar observations apply to the geographic origin of processed samples, although the provenance of specimens from certain datasets (eg IP102) was not considered in this analysis. As shown in Fig. 4, most hyper-biodiverse areas remain undersampled and underrepresented in the published papers.

### Biodiversity and Taxonomy Coverage

This section responds to **RQ1** and discusses the breadth of taxonomic and biodiversity coverage based on the studies analyzed.

#### Biodiversity

Across the reviewed studies, 12 orders were represented, spanning 122 families and 313 genera. The most frequently studied orders were Hemiptera (44 studies), Diptera (43), Lepidoptera (40), and Coleoptera (34). Frequently represented families included Noctuidae (22 studies), Delphacidae (14), Aleyrodidae (14), Curculionidae (13), Chrysomelidae (12), Tephritidae (11), and Aphididae (10). Within these families, a total of 1,634 species were cited cumulatively (non-deduplicated), indicating the current taxonomic reach of AI-based classification. Due to the inclusion and exclusion criteria, adults were the predominant life stage (111 studies), with larvae and eggs being included along with the adult stage in 26 and 9 studies, respectively. From an ecological biodiversity standpoint, this corpus of species represents less than 0.01% of the estimated 5.5 million global insect species. This underscores a strong research bias toward economically important taxa and reinforces the need for broader biodiversity inclusion in future AI-driven entomological research.

The adult stage provides the most accessible stage for image-based recognition. Their defined features, including body segmentation, antennae, and coloration, make them ideal candidates for AI-driven classification. Adult morphology has been extensively used in previous studies to construct accurate classification models. For instance, Wen and Guyer (2012) applied imaging to identify *Cydia pomonella* (L.) (Lepidoptera: Tortricidae), while Pise and Patil (2023) and Park et al. (2020) focused on adult mosquitoes such as *Aedes aegypti* (L.). (Diptera: Culicidae) and *Culex quinquefasciatus* (Say) (Diptera: Culicidae). In the case of *Cnaphalocrocis medinalis* (Guenée) (Lepidoptera: Crambidae), adult-stage recognition enabled by macro photography supported CNN (Convolutional Neural Network)-based classification (Liu et al. 2019). These morphology-based approaches frequently relied on mobile or macro imaging to capture external structures such as body shape, elytral markings, and head capsule dimensions, excluding more complex features like wing venation. This methodology was effective for identifying agricultural pests like *Spodoptera frugiperda* (J.E. Smith) (Lepidoptera: Noctuidae), *Tribolium castaneum* (Herbst) (Coleoptera: Tenebrionidae), and *Sitophilus oryzae* (Linnaeus) (Coleoptera: Curculionidae). High-resolution imaging was central in studies such as Badgujar et al. (2023a,b), Shen et al. (2018), and Gong et al. (2023), which used both lab and field environments to train CNNs on full-body insect images.

Less-used methodologies include spectral imaging, radiographic-based studies, and behavioral tracking with the use of techniques such as macro imaging, wing venation analysis, or behavioral recognition systems (Almryad and Kutucu 2020, Zhang et al. 2024). The interferential patterns present on transparent insect wings were used by a few studies on adult Diptera to differentiate phlebotomine sandflies or mosquitoes (Cannet et al. 2022, 2023a). Behavioral studies analyzed adult flight or thermal behavior using infrared and 3D motion-capture systems, especially in Hymenoptera, such as those performed by Almryad and Kutucu (2020). Methods that used radiographic approaches have been applied to *Sitophilus zeamais* (Motschulsky) (Coleoptera: Curculionidae) in a stored-product environment (Barboza da Silva et al. 2021).

In addition to adults, some publications dealt conjointly with larval and egg stages. 26 studies focused on larvae, which shows the economic interest in including this developmental stage, especially for pest surveillance (Larios et al. 2008, Zhang et al. 2022). In agricultural contexts, larval ­detection of species like *Spodoptera litura* (Fabricius) (Lepidoptera: Noctuidae), *Ostrinia furnacalis* (Guenée) (Lepidoptera: Crambidae), and *Mythimna separata* (Walker) (Lepidoptera: Noctuidae) allowed for timely pest management interventions, with CNNs trained to recognize subtle differences in body segmentation, head capsule morphology, and instar development (Zhang et al. 2022). Other species studied included *Helicoverpa armigera* (Hübner) (Lepidoptera: Noctuidae) and *Maruca vitrata* (Fabricius) (Lepidoptera: Crambidae), which are major pests of cotton, legumes, and cereals. In stored product systems, beetle larvae like *Trogoderma granarium* (Everts) (Coleoptera: Dermestidae) were analyzed using radiographic imaging due to their internal location within grain kernels (Shen et al. 2018, Badgujar et al. 2023a, 2023b). In forest and urban monitoring, larvae of saproxylic beetles were detected using borehole camera systems and spectral reflection models (Tannous et al. 2023). Additional studies employed shape descriptors and fluorescence imaging to enhance differentiation, especially in environments with overlapping developmental stages or low-contrast substrates (Lee et al. 2023, Nasir et al. 2023).

Egg-stage identification appeared in 9 studies, employing hyperspectral imaging, visual proxies, or oviposition behavior inference (Wu et al. 2019, Barboza da Silva et al. 2021, Chen et al. 2021, Hong et al. 2021, Ikeda et al. 2021, Mamdouh and Khattab 2021, Kalfas et al. 2023, Linfeng et al. 2023, Park et al. 2023, Shen et al. 2024). These studies focused on agriculturally important species where early detection is critical to interrupt reproductive cycles. Detection strategies included infrared thermography to visualize embryonic development inside transparent or semi-transparent eggs, as well as backlighting and magnified imaging techniques to classify egg clusters by size, shape, and pigmentation. Some studies inferred egg presence through oviposition behavior captured via time-lapse or motion-triggered imaging. Due to their small size and often cryptic placement on host plants, eggs were among the most challenging life stages to classify, requiring high-resolution imaging setups and, in some cases, the use of advanced computer vision techniques.

Interestingly, 7 studies addressed the entire insect life cycle by including adults, larvae, and eggs (Wu et al. 2019, Barboza da Silva et al. 2021, Ikeda et al. 2021, Mamdouh and Khattab 2021, Kalfas et al. 2023, Linfeng et al. 2023, Park et al. 2023). These integrated approaches aimed to support comprehensive pest surveillance or developmental tracking across stages.

#### Taxonomic Diversity

AI-based insect classification spanned a wide taxonomic spectrum, with applications in pest detection, pollinator monitoring, and vector surveillance. All 111 studies in this review covered representatives from at least 1 of 12 insect orders. The most frequently represented orders were:

##### Hemiptera (44 studies).

This order encompasses many significant agricultural pests and vectors of plant diseases. Key species studied include *Nezara viridula* (Linnaeus) (Hemiptera: Pentatomidae), *Nilaparvata lugens* (Stål) (Hemiptera: Delphacidae), *Sogatella furcifera* (Horváth) (Hemiptera: Delphacidae), *Laodelphax striatellus* (Fallén) (Hemiptera: Delphacidae), and *Bemisia tabaci* (Gennadius) (Hemiptera: Aleyrodidae) (Yu et al. 2022, Linfeng et al. 2023). These insects pose major threats to rice, maize, and vegetable crops in Asia and other regions. AI-based classification systems employed hyperspectral and RGB imaging, often using CNN architectures to distinguish between morphologically similar instars and adult forms. Some studies integrated UAV (Unmanned Aerial Vehicle)-based data acquisition for wide-area pest monitoring in rice paddies (Lee et al. 2023, Linfeng et al. 2023), while others used automated macro photography in greenhouses (Ong and Hamid 2022). Particularly for planthoppers and whiteflies, rapid detection was emphasized due to their ability to transmit viral plant pathogens.

##### Diptera (43 studies).

This order included a wide variety of species with both agronomic and public health relevance. Studies focused on vector mosquitoes such as *A. aegypti*, *C. quinquefasciatus*, *Anopheles stephensi* (Liston) (Diptera: Culicidae), and *Culex pipiens* (Linnaeus) (Diptera: Culicidae), which are critical in disease surveillance for dengue, malaria, and filariasis (Mamdouh and Khattab 2021, Pise and Patil 2023). Biting midges such as *Culicoides imicola* (Kieffer) (Diptera: Ceratopogonidae) and *Culicoides obsoletus* (Meigen) (Diptera: Ceratopogonidae) were studied for their role in transmitting livestock viruses like bluetongue (Ikeda et al. 2021). Vector species such as *Glossina morsitans* (Westwood) (Diptera: Glossinidae) (tsetse fly) (Barboza da Silva et al. 2021) and *Phlebotomus* sp. (phlebotomine sandflies) (Cannet et al. 2023a) were also included due to their importance in human and veterinary health. Other significant species studied include *Lucilia sericata* (Meigen) (Diptera: Calliphoridae) (Ikeda et al. 2021), *Chrysomya megacephala* (Fabricius) (Diptera: Calliphoridae) (Ikeda et al. 2021), *Sarcophaga carnaria* (Linnaeus) (Diptera: Sarcophagidae) (Ikeda et al. 2021), and *Calliphora vicina* (Robineau-Desvoidy) (Diptera: Calliphoridae) (Chen et al. 2021), which are relevant to forensic entomology and decomposition studies. Tephritid fruit flies such as *Bactrocera* and *Ceratitis* were also frequent subjects, reflecting their agricultural relevance (Ong and Hamid 2022, Tannous et al. 2023). Species from the genera *Drosophila* (Kalfas et al. 2023) and *Musca* (Hong et al. 2021) were investigated for developmental biology and urban pest modeling. These species were analyzed in both lab and field scenarios, with several studies incorporating CNNs into automated traps and mobile apps for real-time vector surveillance, forensic casework, and habitat assessment.

##### Lepidoptera (40 studies).

Studies of this order included pests such as *S. frugiperda* (J.E. Smith) (Lepidoptera: Noctuidae) (Zhang et al. 2022), *C. medinalis* (Guenée) (Lepidoptera: Crambidae) (Park et al. 2020), *C. pomonella* (Wen and Guyer 2012, Preti et al. 2021), *H. armigera* (Linfeng et al. 2023), *Conogethes punctiferalis* (Guenée) (Lepidoptera: Crambidae) (Preti et al. 2021), *Argyrotaenia velutinana* (Walker) (Lepidoptera: Tortricidae) (Preti et al. 2021), *Earias insulana* (Boisduval) (Lepidoptera: Nolidae), *Sesamia inferens* (Walker) (Lepidoptera: Noctuidae), *Anticarsia gemmatalis* (Hübner) (Lepidoptera: Erebidae), *M. vitrata* (Zhang et al. 2022), *Heliothis virescens* (Fabricius) (Lepidoptera: Noctuidae) (Ikeda et al. 2021), and *Antheraea pernyi* (Guérin-Méneville) (Lepidoptera: Saturniidae) (Wu et al. 2019, Barboza da Silva et al. 2021, Chen et al. 2021, Hong et al. 2021, Ikeda et al. 2021, Mamdouh and Khattab 2021, Kalfas et al. 2023, Linfeng et al. 2023, Park et al. 2023, Shen et al. 2024). For instance, *Spodoptera*, *Helicoverpa*, and *Mythimna* are major threats to cereal crops and cotton, while *Plutella* targets brassicas and is a critical pest in apple orchards. On the biodiversity side, *A. pernyi*, a silk moth, and *Argyrotaenia* species are monitored for ecological impact assessments. Nevertheless, these genera and species were studied for their economic impact on crops like maize, rice, cotton, and fruit trees across Asia, Europe, and the Americas. Several of these, such as *Spodoptera* and *Helicoverpa*, were monitored at multiple developmental stages, using smart traps or pheromone-lured devices integrated with CNNs and object detection models. Others, like *Plutella* and *Argyrotaenia*, were studied for their early larval identification, essential for preventing infestations in brassicas and orchards. Lepidoptera were also among the few orders for which multiple life stages were studied (adults, larvae, and eggs) due to their significance in agriculture and their role in ecosystem dynamics.

##### Coleoptera (34 studies).

This order was prominently represented by studies targeting stored-product beetles and field pests. Economically important species included *T. castaneum* (Shen et al. 2018, Badgujar et al. 2023b), *S. oryzae* (Shen et al. 2018), *S. zeamais* (Badgujar et al. 2023a, 2023b), *Rhizopertha dominica* (Fabricius) (Coleoptera: Bostrichidae) (Badgujar et al. 2023b), *Oryzaephilus surinamensis* (Linnaeus) (Coleoptera: Silvanidae) (Shen et al. 2018), *T. granarium* (Shen et al. 2018), *Lasioderma serricorne* (Fabricius) (Coleoptera: Ptinidae) (Badgujar et al. 2023b), and *Bruchus pisorum* (Linnaeus) (Coleoptera: Chrysomelidae) (Nasir et al. 2023). These beetles are major threats to post-harvest grain and food processing systems. Techniques ranged from radiograph-based deep feature extraction to mobile phone-enabled camera classification systems. In addition to stored-product beetles, some studies included field beetles from families like Coccinellidae and Chrysomelidae to evaluate broader pest and predator roles. Coccinellids, such as *Coccinella septempunctata* (Linnaeus) (Coleoptera: Coccinellidae) (Almryad and Kutucu 2020), were included in datasets exploring multi-species interactions, particularly in agricultural biodiversity assessments. Applications of AI in Coleoptera emphasized precision pest monitoring, early detection in storage systems, and ecological modeling of species interactions in crop ecosystems (Shen et al. 2018, Badgujar et al. 2023b).

##### Hymenoptera (21 studies).

Studies of this order included social insects of major ecological and agricultural importance. Key taxa studied were *Apis mellifera* (Linnaeus) (Hymenoptera: Apidae) (Chen et al. 2021), *Bombus terrestris* (Linnaeus) (Hymenoptera: Apidae) (Kalfas et al. 2023), *Vespa velutina* (Lepeletier) (Hymenoptera: Vespidae) (Kalfas et al. 2023), *Vespa orientalis* (Linnaeus) (Hymenoptera: Vespidae) (Kalfas et al. 2023), and several species of ants (Formicidae), particularly within the genera *Camponotus* and *Solenopsis* (Almryad and Kutucu 2020). Applications ranged from pollination ecology to invasive species monitoring and interspecies interactions. AI systems were used to classify bee and hornet behavior around hives, track flight trajectories using 3D cameras and infrared sensors, and monitor pollinator activity in orchards and open habitats. Hornets were often tracked to assess their predation patterns on bees, while *A. mellifera* was the subject of vibration and image-based monitoring for colony health. Ants were studied for colony structure and behavior recognition using video classification and thermal imaging.

##### Orthoptera (13 studies).

Studies of this order included agriculturally significant pests such as *Gryllotalpa* spp. (Orthoptera: Gryllotalpidae) (mole crickets), *Atractomorpha sinensis* (Bolívar) (Orthoptera: Pyrgomorphidae) (gaudy grasshoppers), and *Teleogryllus emma* (Ohmachi and Matsuura) (Orthoptera: Gryllidae) (field crickets). These insects are known for damaging root systems, stems, and leaves in rice, horticultural, and cereal cropping systems, among others. Verified studies include *Gryllotalpa* spp. (Hong et al. 2021) and *A. sinensis* (Wang et al. 2020). AI-based classification in these studies relied on visual recognition techniques, primarily macro imaging and object detection applied to field-captured specimens.

##### Thysanoptera (13 studies).

Studies of this group primarily focused on *Scirtothrips dorsalis* (Hood) (Thysanoptera: Thripidae), a highly invasive pest of citrus and fruit crops. Detection systems were developed using smartphone-based DL applications, which achieved real-time classification performance (Niyigena et al. 2023). *Drepanothrips reuteri* (Uzel) (Thysanoptera: Thripidae) was also included in multi-species detection tasks, particularly in field imaging conditions with embedded AI systems (Park et al. 2023).

##### Blattodea (formerly Isoptera) (4 studies).

Termite classification focused on economically significant species such as *Cryptotermes domesticus* (Haviland) (Blattodea: Kalotermitidae) (Huang et al. 2021), *Coptotermes formosanus* (Shiraki) (Blattodea: Rhinotermitidae) (Huang et al. 2021), *Reticulitermes flaviceps* (Oshima) (Blattodea: Rhinotermitidae) (Chen et al. 2021), and *Odontotermes formosanus* (Shiraki) (Blattodea: Termitidae) (Huang et al. 2021). A dataset of over 24,000 caste-specific images (soldiers vs. workers) was compiled, enabling the development of mobile surveillance applications.

##### Dermaptera (3 studies).

Across the 3 studies, Dermaptera was represented by earwigs from the family Forficulidae, recorded in the HI30 hyperspectral dataset with 74 samples but identified only to family level rather than species (Tan et al. 2024). In the multispectral machine vision work, unspecified species of earwigs were among 12 invertebrate pests detected on green leaves under field conditions, demonstrating the potential of ultraviolet, visible, and near-infrared imaging for distinguishing them from foliage (Liu and Chahl 2018). Together, these studies indicate that Dermaptera were largely represented only at the family level (Forficulidae) or described generically as “earwigs,” highlighting the limited taxonomic resolution currently available for this order in existing datasets. However, an exception is provided by Gomes et al. (2023), who included the dermapteran species *Doru luteipes* as a distinct class in a maize insect dataset, demonstrating that species-level annotation is achievable for this order in applied agricultural contexts. Nonetheless, most other studies still represent Dermaptera only at the family level (Forficulidae) or as generic “earwigs,” indicating that high-resolution taxonomic representation remains uncommon.

##### Mantodea (3 studies).

Mantodea, including species such as *Mantis religiosa* (Linnaeus) (Mantodea: Mantidae) and other crop-associated mantids, were used as reference taxa in recent AI-based insect recognition research (Cao et al. 2020). Multi-scale convolutional attention networks further improved discrimination among morphologically similar taxa, highlighting the potential of fine-grained feature fusion to recognize mantids under complex field conditions (Wang et al. 2024). Complementary CNN frameworks integrating preprocessing and large-scale image datasets reinforced the feasibility of recognizing mantids as part of broader pest and natural enemy assemblages, reducing reliance on manual identification and supporting precision agriculture (Linfeng et al. 2023).

##### Odonata (2 studies).

Dragonflies and damselflies from families like Platycnemididae and Gomphidae were studied primarily as bioindicators in freshwater ecosystem monitoring. AI models trained on camera trap and habitat image data recognized these taxa based on their wing posture, color morphology, and body shape, supporting their use in water quality assessments and biodiversity tracking frameworks (Wang et al. 2020, Tan et al. 2024).

##### Neuroptera (1 study).

Lacewings (Neuroptera: Chrysopidae, eg *Ceraeochrysa*) were investigated for their taxonomic characterization within maize agroecosystems (Gomes et al. 2023), where their role as beneficial predators is well recognized. The study demonstrated that species-level identification based on morphological traits such as wing venation, head–thorax shape, and color patterns can be effectively captured from field images, allowing reliable discrimination among closely related taxa. This approach achieved high classification accuracy even with limited training data, underscoring the diagnostic value of key morphological features for neuropteran taxonomy (Gomes et al. 2023).

##### Mecoptera (1 study).

Mecopteran scorpionflies (Mecoptera: Panorpidae, eg *Panorpa*) were included to evaluate taxonomic recognition within automated insect imaging and classification workflows (Brandt et al. 2024). Their distinctive elongated rostrum, patterned wings, and characteristic abdomen shape make them visually salient, enabling reliable discrimination from other insect orders in multi-taxon computer vision pipelines despite limited training data (Brandt et al. 2024).

### Uneven Representation Across Insect Taxa

While DL and computer vision methods have been applied across a wide range of insect taxa, the reviewed studies revealed a strong taxonomic bias. Most research focused on a limited number of economically or medically significant orders, particularly Lepidoptera, Diptera, Hemiptera, and Coleoptera, reflecting pest management and vector surveillance priorities. In contrast, ecologically important but less economically prioritized groups, such as Odonata, Blattodea, Orthoptera, Dermaptera, Mantodea, and Neuroptera, appeared only sporadically, often as auxiliary taxa or part of multi-order datasets.

Several insect orders, including Phasmatodea, Plecoptera, Trichoptera, Ephemeroptera, and Psocoptera, were entirely absent from the reviewed literature, despite their ecological and bioindicator relevance. This absence highlights the narrow taxonomic scope of current AI-based entomological studies. This uneven representation limits the generalizability of existing AI models and hampers their applicability to biodiversity monitoring, ecosystem assessment, and early detection frameworks. Expanding research to include neglected insect orders will be essential for developing more inclusive and ecologically relevant AI-driven insect classification systems.

### Experimental Setup and Optical Devices

An important dimension in AI-based insect classification is the setting in which data are collected: field versus laboratory environments. The different imaging systems and data acquisitions methods are covered in this section and address **RQ2**.

#### Experimental Setting

##### Field-based studies.

Field studies offer the advantage of ecological validity. These typically utilized smart traps, sticky traps or pheromone traps, and mobile imaging systems to gather real-time or in situ data on insect behavior, distribution, and phenology. Examples include works of Wen and Guyer (2012) and Preti et al. (2021) for *C. pomonella*, Gerovichev et al. (2021), which use sticky traps for *Thaumastocoris peregrinus* (Carpintero and Dellapé) (Hemiptera: Thaumastocoridae), and Liu et al. (2019) for *C. medinalis* (Guenée) (Lepidoptera: Crambidae). Zhang et al. (2022) monitor *M. separata* and *H. armigera* via pheromone traps. Field deployments inherently involved variability in lighting, background clutter, and insect orientation. Models trained in these environments require robustness and were often complemented by data augmentation or domain adaptation techniques to handle unpredictable inputs.

##### Laboratory-based studies.

Laboratory-controlled studies emphasized high-resolution imaging, precise labeling, and environmental consistency. These studies included imaging stored-product pests like *T. castaneum*, *O. surinamensis*, and *T. granarium* (Shen et al. 2018, Badgujar et al. 2023b), and larval stages of *S. litura*, *M. separata*, and *M. vitrata* (Zhang et al. 2022). Ant behavior and colony interactions were evaluated in controlled conditions with *Solenopsis* and *Camponotus* (Almryad and Kutucu 2020). Thrips recognition models including *S. dorsalis* were developed in lab-based lighting simulations by Niyigena et al. (2023). Cannet et al. (2023a,2023b) applied interferential wing pattern analysis for adult Diptera. Additional lab-focused studies such as (Kalfas et al. 2023) worked on *B. terrestris* and *V. velutina*, while Nasir et al. (2023) developed controlled datasets for *B. pisorum*. Lab studies typically yield higher-quality datasets and greater control over life stage selection and taxonomy but may lack the ecological noise that field-trained models must accommodate with. Consequently, the transferability of laboratory-trained models to real-world conditions remains a central challenge for AI-based entomology.

##### Hybrid approaches.

Some studies combined field and laboratory components, capturing wild insects in natural settings and subsequently imaging them under controlled laboratory conditions. This strategy allows researchers to balance ecological realism with experimental precision, enabling high-quality datasets that still reflect natural variation. For instance, field-collected ants were re-imaged under standardized lighting to study behavioral interactions in *Solenopsis* and *Camponotus* (Almryad and Kutucu 2020). Similar workflows were employed in other studies to improve annotation quality and control background noise while preserving authentic morphological diversity. Such hybrid methodologies offer a practical compromise between the variability of field environments and the reproducibility of laboratory experiments, though harmonizing data across acquisition contexts remains a technical challenge.

#### Trapping Methodologies

##### Smart/image-based traps.

Smart traps are autonomous data acquisition systems equipped with embedded cameras, often enhanced by onboard processing units running CNNs or other machine learning (ML) algorithms. These devices capture real-time images or videos of insects directly in the field, enabling automated identification, counting, and behavior monitoring. Common deployment environments include orchards, rice fields, grain storage units, and greenhouse systems. Preti et al. (2021) developed a smart trap for orchard moth monitoring using an embedded system that captured and classified *C. pomonella* and *A. velutinana*. Wen and Guyer (2012) pioneered a similar orchard-based image collection for tortricid moths. Chen et al. (2021) used smart traps to detect the yellow stem borer, *Scirpophaga incertulas* (Walker) (Lepidoptera: Crambidae). Shen et al. (2018) created a camera-based trap for stored-product beetles, with automated recognition of species like *T. castaneum*, *O. surinamensis*, and *T. granarium*. Simulated smart trap conditions were also developed to classify beetle species such as *S. zeamais*, *R. dominica*, and *L. serricorne* under controlled lighting scenarios (Badgujar et al. 2023a).

##### Sticky traps.

Sticky traps captured flying pests in crop and forest ecosystems. They were often combined with macro imaging and CNNs (Park et al. 2020, Gerovichev et al. 2021, Lee et al. 2023).

##### Pheromone traps.

Pheromone traps employed species-specific chemical lures to attract target insects, primarily male moths, making them particularly effective for population monitoring and integrated pest management (IPM) programs. These traps were especially prevalent in Lepidoptera research, aiding early detection and classification of key pest species such as *S. frugiperda* (Ong and Hamid 2022), *M. separata* (Zhang et al. 2024), and *C. pomonella* (Wen and Guyer 2012, Preti et al. 2021).

##### Light traps.

Light traps were used in 5 studies, each focusing on nocturnal or crepuscular insect species of agronomic importance. Liu et al. (2019) deployed a searchlight-based trap with video cameras to monitor *N. lugens* (brown planthopper) in rice paddies. Linfeng et al. (2023) integrated light traps with image-based CNNs for detecting *M. separata* (Walker). Almryad and Kutucu (2020) used lighting systems to study behavioral responses in *Empoasca fabae* (Harris) (Hemiptera: Cicadellidae).

##### Simulated/visual traps.

Simulated or visual traps were used to generate consistent datasets for AI training when field data were scarce. De Geus et al. (2019) constructed an endoscopic chamber to simulate maize environments, supporting pests classification, like *S. zeamais*. Shen et al. (2018) created simulated grain trap environments for stored-product beetles like *T. castaneum* and *T. granarium*. Park et al. (2023) developed a lab-based setup using printed visuals of *S. dorsalis* on leaves for smartphone detection. Additional examples include Ikeda et al. (2021), who modeled visual traits of blowflies like *L. sericata* under controlled illumination; Kalfas et al. (2023), who generated controlled visuals for *B. terrestris* detection; and Nasir et al. (2023), who simulated backgrounds to enhance recognition of *B. pisorum*. Lastly, Niyigena et al. (2023) built image-based simulations to detect *S. dorsalis* under varied light intensities.

#### Optical Devices

**Fig. 5.**
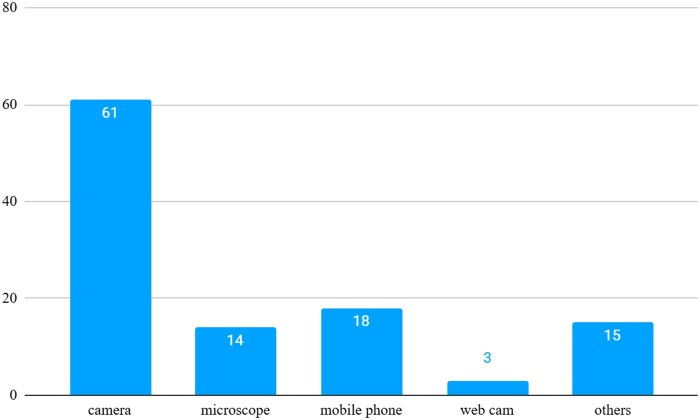
Devices used for image capture, including professional cameras, microscopes, mobile phones, webcams, and other imaging systems.

A variety of image capture devices were employed in the 111 studies that were shortlisted, as illustrated in Fig. 5, including professional cameras, microscopes, mobile phones, webcams, and a few other image acquisition devices. In some cases, these devices were complemented with additional tools such as light sources or magnifiers to enhance image quality. The choice of imaging method largely depended on the data collection context, distinguishing between field and laboratory settings.

Professional cameras, used in 61 studies, and mobile phones, used in 18 studies, have been used mainly in field settings, such as for live detection of pests (Rimal et al. 2023) and for the identification of the fall armyworm in maize fields (Zhang et al. 2022). Fifteen studies used only a mobile phone as the image-capturing device (Huang et al. 2021, Cochero et al. 2022, Rimal et al. 2023). At least 1 study used a magnifier along with the mobile phone, namely Li et al. (2023), who used a 200× macro lens with built-in LED light. Sixty-one studies used high-definition cameras for image acquisition. For instance, Zhang et al. (2022) photographed maize pests such as the fall armyworm (*S. frugiperda*), Tannous et al. (2023) captured images of economically important insect species, including pest flies, and Zhu et al. (2017) used a Nikon D200 digital camera for imaging lepidopteran insects.

Fourteen studies made use of a microscope for image capture, mainly carried out in controlled environments. For example, Wen and Guyer (2012) used a Nikon DS-Fi1 color digital camera mounted on a Nikon stereoscopic zoom microscope to capture insect images. Guerron et al. (2016) used a Zeiss V20 microscope coupled with a Sony DSC camera to capture Diptera wing images. Fujisawa et al. (2023) individually captured a subset of a single specimen using a conventional stereoscope NIKON SMZ1270i equipped with a NIKON DS-Fi3 microscope camera controlled by the NIKON DS-L4 control unit. Additionally, most of these studies involved mounting slides for capturing the images, like those performed by Cannet et al. (2022, 2023a, 2023b) to capture WIP on wings deposited on a glass slide and covered by a small cover slide with a Keyence VHX 1000 microscope and a VH-Z20r lens.

Three studies used a webcam as an image capture device. For example, Bjerge et al. (2021) used a Logitech webcam with a resolution of 3840 × 2160 pixels in their Automated Moth Trap, while Wen and Guyer (2012) utilized a webcam for continuous image capture in a flight tunnel to monitor insect behavior. Similarly, Hadi et al. (2021) employed a camera-based imaging system for automated pest sampling and detection using motorized sticky traps.

Several studies incorporated specialized lighting systems to enhance image quality. For instance, Sun et al. (2018) used a digital camera with a 2.1 mm prime lens, accompanied by an LED light band for optimal exposure; Hong et al. (2021) employed a color Sony camera integrated into a photographing system equipped with 4-way LED lighting; and Liu et al. (2022) developed an image acquisition platform comprising an imaging system, insect body transmission system, sensors, light sources, and dedicated image acquisition software. Wen and Guyer (2012) used a Gooseneck light guide powered by a Schott-Fostec Eke Pheostat 150 W light source. Other studies used only a camera with LED lighting (Sun et al. 2018, Hong et al. 2021).

Interestingly, 15 studies used other image acquisition devices. Four studies used an Internet of Things(IoT)-based camera system. Ramalingam et al. (2020) employed an IoT-based Trek AI ball WiFi-enabled camera, which could be fixed on the roof of large food storage containers for continuous insect monitoring; Sittinger et al. (2024) used a solar-powered IoT-based camera along with a Raspberry Pi Zero 2 W system to capture images of insects landing on a specific platform; Preti et al. (2021) used an IoT-based Omnivision OV5648 camera system for remote monitoring of insect pests; and Sun et al. (2022) utilized an IoT-based Omnivision OV5648 camera attached to a stepper motor to automatically capture images in a greenhouse environment.

Other studies employed a variety of specialized imaging devices. Park et al. (2023) used a GoPro Hero 8 Black camera mounted on a ground vehicle, while Li et al. (2021) utilized an image-acquisition device developed by the National Engineering Research Center for Information Technology in Agriculture to capture images of pests on a yellow sticky trap. Barboza da Silva et al. (2021) employed a MultiFocus instrument integrated with a CMOS X-ray sensor for capturing X-ray images. Tuda and Luna-Maldonado (2020) used an Epson GT-X820 scanner to image specimens placed in random orientations within a clear plastic box. Yang et al. (2023) implemented a distributed field data acquisition system using CCTV cameras equipped with 100-W illumination, generating 25,222 static images to document activities and damages caused by pests on grain surfaces.

Even if various devices were used to capture images, professional cameras were the most popular option. Examples included Canon EOS 700D, Olympus camera E-M10, DSC-H300 camera, and the VH-Z20r lens amongst many others. High-resolution cameras provided superior image quality, which made the subsequent insect detection and classification tasks easier. As highlighted by Bisgin et al. (2022), prediction accuracy is strongly influenced by the quality of the captured images. Hence, studies using professional cameras generally achieved higher accuracy rates, such as 99.30% in Xiong et al. (2024) and 99.1% in Dai et al. (2020), although Wang et al. (2024) reported a lower accuracy of 92.36%. In contrast, studies utilizing mobile phones as imaging devices yielded slightly lower accuracy, for example, 95% (Li et al. 2023), 93.3% (Niyigena et al. 2023), and 90.2% (Dai et al. 2021). Several researchers enhanced image quality by incorporating external light sources (Tan et al. 2024) or magnifiers (Li et al. 2023).

Cameras and mobile phones have been predominantly used for field imaging, while microscopes were preferred in controlled laboratory environments (Guerron et al. 2016, Park et al. 2020). Although mobile phones can produce high-quality images, their use in entomological applications remains challenging, particularly under field conditions, where insect movement is difficult to control. Future studies, comparing images captured by cameras and mobile phones under controlled conditions, might allow the researchers to move toward cheaper solutions for image capture.

An almost even distribution was observed between studies conducted under laboratory-controlled settings and those based on field-acquired data. Laboratory settings offered high-quality imaging, consistent lighting, and precise labeling—conditions well suited for model development and initial testing. However, models trained exclusively in such environments often face difficulties when applied in real-world scenarios, where variations in lighting, background complexity, and insect orientation can reduce robustness. Field-based studies, while offering greater ecological validity, are typically affected by noise, uneven sampling, and data imbalance. A few hybrid approaches have attempted to bridge this gap by capturing field specimens and imaging them under controlled conditions or by using data augmentation to simulate field variability. Moving forward, progress in domain-adapted models and field-compatible imaging systems is essential to ensure generalizability and operational scalability of AI-based classification tools.

### Datasets

The different insect image datasets used in this study are described in this section and address RQ3.

**Fig. 6.**
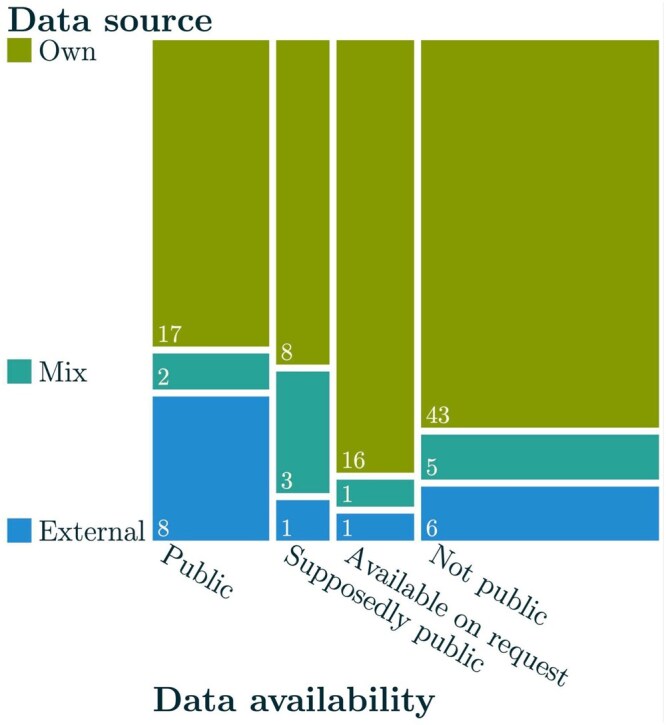
Breakdown of the 111 papers based on the source of the data source used (own acquisitions, data from others or a mix) and on the availability of the data as provided by the paper: public, supposedly public (includes broken links), data Available On Request (AOR), or not public.

Figure 6  gives an overview of both the data sources of the papers reviewed and their availability. A vast majority of the studies relied solely on their own data acquisitions or previous acquisitions from co-authors (84 out of 111). Only 16 studies solely rely on external data, while 11 studies use a mix of external data and own acquisitions. Public datasets provided or used by the reviewed papers are listed in Table 1. The public links to the datasets are available in Supplementary Material S3.

**Table 1.** Overview of public datasets used in the papers reviewed, grouped as Maintained, Open-Application Datasets (MOAD) and Application-Specific Datasets (ASD)

Name	Reference	Scope	Number of images	Used outside original team
**AdaMerOs-Butterflies of Turkey**	http://adamerkelebek.org	MOAD	671,888 images	✔✔️
**Bugwood Image Database**	https://images.bugwood.org/	MOAD	331,811 images	✔✔️
**iNaturalist**	https://www.inaturalist.org/	MOAD	>76,000,000 images	✔✔️
**IP102**	Wu et al. 2019	MOAD	75,000 images	✔✔️
**National Bureau of Agricultural Insect Resource**	https://www.nbair.res.in/databases	MOAD	Unknown (database repository)	✔✔️
**SITE-100**	Bian et al. 2022	MOAD	34,301 images	✔✔️
**Waarnemingen**	https://waarnemingen.be/	MOAD	Unknown	✔✔️
***Aedes* Mosquitos Dataset**	Ong et al. 2021	ASD	4,120 images	✔✔️
**Dataset of Vector Mosquito Images**	Pise et al. 2022	ASD	44 images	✔✔️
**Inventory of Butterfly Species of Sangay National Parc**		ASD	2,799 images	✔✔️
**Leeds Butterfly dataset**	Wang et al. 2009	ASD	832 images	✔✔️
**MosquitoDL**	Park et al. 2020	ASD	3,600 images	✔✔️
**BEE4EXP**	Stojnić et al. 2021	ASD	6 videos	
**BPH and BENIGN**	Nazri et al. 2018	ASD	122 images	
**Brown Marmored Stink Bug Dataset**	Kargar et al. 2024	ASD	687 images	
**Bulk images**	Fujisawa et al. 2023	ASD	2,754 images	
**Diptera WIPS**	Sereno et al. 2024	ASD	5,516 images	
**DL-termite-identification**	Huang et al. 2021	ASD	24,000 images	
***Drosophila melanogaster* dataset**	Schneider et al. 2018	ASD	180 videos as image frames	
**GeoVin**	Cochero et al. 2022	ASD	563 images	
**Honeybee video tracking data**	Ratnayake et al. 2021	ASD	7 video sequences and 2,799 images	
**Insect Detect classification**	Sittinger et al. 2024	ASD	21,000 images	
**Insect Detect training**	Sittinger et al. 2024	ASD	1,335 images	
**Ladybird beetle images**	Venegas et al. 2021	ASD	2,300 images	
**Maize crops few-shot insect dataset**	Gomes et al. 2023	ASD	420 images	
**MCC-trap**	Bjerge et al. 2021	ASD	2,000 images	
**Medical and forensically important flies**	Ong and Hamid 2022	ASD	2,875 images	
**NEON carabid dataset**	Blair et al. 2020	ASD	3,265 images	
**TF4**	Shen et al. 2024	ASD	3,409 images	
**Vegetation pests and diseases data set**	Linfeng et al. 2023	ASD	15,905 images	

Only datasets with a functioning link with access to data are included in this table.

We sorted public datasets in 2 categories: Maintained Open-Application Datasets (MOAD) and Application-Specific Datasets (ASD). MOADs tend to be larger and built with a wide scope of uses in mind; it is also important to note that they seem to be actively maintained. ASDs, on the other hand, are public datasets that were built by a group of collaborators with their specific work in mind. Though the separation between the 2 categories may not be clear-cut (a dataset may switch from 1 to the other over time), this helps grasp the scope of the datasets.

The most commonly used MOADs (large and publicly available datasets) are IP102 (Wu et al. 2019) and the Bugwood Image Database (Bugwood 2024) (through the Bugwood website, Insect Images [2024], or IPM Images), and iNaturalist (iNaturalist 2024), which is more generic and includes over 760,000,000 images of Insecta.

The studies that leveraged these datasets spanned a wide taxonomic diversity, with Lepidoptera, Coleoptera, Hemiptera, and Hymenoptera particularly well represented. Their datasets contained tens of thousands of labeled images across more than 100 species, making them valuable for general-purpose recognition tasks. The works of Murali et al. (2019), Wu et al. (2019), Ramalingam et al. (2020), Amrani et al. (2023), Rimal et al. (2023), and Kathole et al. (2024) illustrate how such benchmarks enable DL pipelines trained on images captured mainly with standard cameras in field conditions. Their scale and diversity allowed cross-validation and provided the community with reference points for insect classification research.

The 95 studies that acquired their own data provide 17 public datasets (ASD), which often focus on narrower ecological or agricultural contexts. For example, the Maize Crop few-shot Insect Dataset (Gomes et al. 2023) covered pests from Hemiptera, Lepidoptera, Coleoptera, and Hymenoptera families, with images collected using field cameras. Similarly, Park et al. (2020) shared curated datasets dedicated to certain pest insects, while Cochero et al. (2022) and Sittinger et al. (2024) provided targeted collections of bees or flies. These datasets, although smaller in scale compared to IP102, frequently employed Digital Single-Lens Reflex or mobile phone cameras under natural lighting, and were tailored to specific pest monitoring or pollinator recognition applications.

A few of these datasets are reused outside the original team. Asgari et al. (2022) provide a notable example of data reuse from various public Mosquito databases: MosquitoDL (Park et al. 2020), Dataset of Vector Mosquito Images (Pise et al. 2022), and Aedes Mosquitos Dataset (Ong et al. 2021).

Beyond the public datasets, 12 papers were classified as having “supposedly public” data due to broken links (4 cases), approval-restricted access (2 cases each via Google Drive or Baidu), or claims that data would be made public although no link was available during this review. All 4 datasets with links that are currently broken have been reused by other teams, showing their global usefulness. PlantVillage (https://arxiv.org/abs/1511.08060), in particular, no longer provides data at the original link, but the data has been copied to other repositories by other teams. This highlights the importance of carefully selecting a data repository at the time of publication for wide reuse over time. Finally, we note that 18 studies included a statement that “Data is available upon request.” This practice does not align with the Findability, Accessibility, Interoperability, and Reusability principles (Wilkinson et al. 2016).

These resources are regionally concentrated, mostly in China, North America, and Europe, which highlights a global imbalance. Notably, tropical and hyper-diverse regions, such as sub-Saharan Africa and parts of Southeast Asia, are significantly underrepresented. This disparity stresses the need for broader data-sharing practices and targeted investment in dataset development from biodiversity-rich areas to support equitable and globally relevant entomological research.

## Computational Methodologies for Image-Based Insect Classification and Identification

This section addresses **RQ4** and reports the different computer vision algorithms identified from the studies.

### Methodologies

As a consequence of the selection process, all considered articles tackled the same type of meta task, which was to extract relevant information from images about the presence or absence of insects and their identification.

We thus propose the following post-acquisition processing framework to analyze the papers:

- Image or data pre-processing, denoising, for example.
- Detection, automatic selection of Regions of Interest (ROIs).
- Segmentation, eventual per-pixel masking of the ROIs.
- Identification, which corresponds to labeling each ROI.

Not all of the 4 steps were present in the 111 articles selected, depending on the focus of the paper.

On the overall set of selected papers, 4 types of approaches were used to tackle the objectives described above:

- Low-level image processing, including, for example, thresholding on gray level value or color channel;
- shallow ML approaches often based on hand-crafted features, such as SIFT (scale-invariant feature transform) or HOG (histogram of oriented gradients) jointly with classic ML method (K-Nearest Neighbor—KNN, Support Vector Machine—SVM, Random Forest);
- full DL approaches (with different types of architectures); and finally
- a mix of (ii) and (iii).

**Fig. 7.**
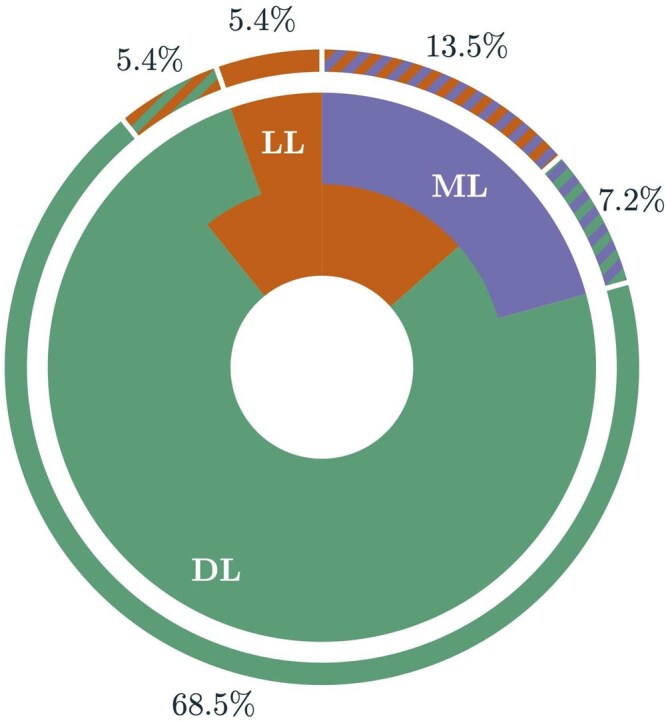
Percentage of articles based on feature extraction or detection method (inner circle) and classification method (middle circle) among Deep Learning (DL), Machine Learning (ML) or Low-Level features (LL). Quantitative values are given on the outer circle.

Figure 7 summarizes the findings.

Low-level image processing often remains a useful step to enhance specific features (colors, patterns, etc.) to feed ML or DL algorithms. It is also used to better frame the image on the insect, or on the relevant part of the insect, in order to avoid distractions from surrounding regions that could be considered as noise. This type of approach was used in 6 papers, in particular, when non-conventional acquisitions are considered, including Near Infrared, Ultra violet, or Spectral Imaging. It helps in extracting simple features that, in the context of the application targeted, are sufficient to detect insects. Nevertheless, it is still limited in terms of performance as soon as the task becomes more complex or the application case less specific.

It is also interesting to notice that shallow ML is still gathering some interest mainly from entomologist or biologist experts. The originality of the research work falling in this category lies in their ability to conjugate hand-crafted feature extraction with statistical learning tools. Kalmann, Gabor filters, HOG, and SIFT are the most used descriptors jointly used with KNN, SVM (with Gaussian kernel mainly), or Decision trees. SVM remains the preferred option but in some cases “Bag of features” approach or boot strapping are used. Their main advantage is the interpretability by experts of obtained results, since the classification can easily be visualized in 2 or 3 dimensions using dimension reduction strategies such as Principal Component Analysis or Linear Discriminant Analysis. These methods can also be easily implemented using Malab© or R© and are not very demanding in terms of computational resources or even data to be fed with, while their level of performance remains adequate for well-defined classification tasks.

DL methods have, unsurprisingly, gathered most of the attention over the last 10 years. Over 80% of the papers used deep architectures for varying tasks, from detection to identification. For detection, the YOLO (You Only Look Once) architecture (v2, v3, v4, and v5) was the most used method (not far from 25% of the papers using Deep-based approaches). The fact is that beyond very good performance, the method can be easily implemented using the Python library and also exists in a tiny version, which is less demanding in terms of computational resources. YOLO is also sometimes used for identification, but in this case, a high level of annotation in terms of classes and confidence is required. YOLO also has the advantage of being real-time compatible with a classic camera frame rate if needed for in natura use.

For the classification/identification task, apart from YOLO as an end-to-end single-architecture solution for the overall processing scheme, it is interesting to notice that the classic historical CNN architectures are mainly considered by entomologists or biologists: ResNet, VGG (Visual Geometry Group), EfficientNet, AlexNet, and GoogleNet, were often cited and considered as an alternative (in the given order) as well as for performance comparison. These architectures have long been easily available on the Python Library and can be used in several scenarios: learning from scratch and transfer learning with pretrained available weights (usually pretrained on the ImageNet data set). MobileNet was also used as a classic architecture with the possibility to embed it easily in smartphone or smart cameras: 23% of the papers using DNN considered this architecture either as their proposition or as a comparison with others. The Recurrent Neural Network option was also considered when the analysis needed to consider the sequence or contextual information surrounding the insect or region of interest. This approach allowed the model to refine its detection by “looking around” the focal area, improving accuracy in complex images. The optimized Faster R-CNN (Region-based CNN) version was the most used in that case, with 12% of the papers based on Deep approaches.

DNN was also considered in a few papers for a segmentation task whose objective was to morphologically characterize parts of the insects or related area of location. Unet has been used in this case, despite the fact that it is a demanding option in terms of computational resources.

Over 7% of the papers proposed to mix shallow classification with DNN feature extraction. The most popular combinations were ResNet + SVM, YOLO + K-means followed by various combinations of AlexNet and VGG with SVMs and KNNs.

Also, Generative Adversarial Networks (GANs) are an emerging option for data augmentation (2 papers focus on this specific task). The databases usually considered for this review work were highly heterogeneous, not necessarily available and possibly unbalanced when considering a large number of insect species. Data generation appeared as an option to overcome the challenge of small data sets.

Finally, Gradient-weighted Class Activation Mapping (Grad-CAM) (Selvaraju et al. 2017), and to a lesser degree CAM (Zhou et al. 2016), were used in over 10 papers. In 2 cases (Bollis et al. 2020, Zhang et al. 2022), Grad-CAM was used as a saliency map to extract features. In all the other cases, these networks were used to visualize the features on which the deep network is based and provide a basis of explainability.

The need for explainability and the bottlenecks provided by the datasets were 2 specificities of insect recognition.

### Performance Criteria and Evaluation Methods

**Fig. 8.**
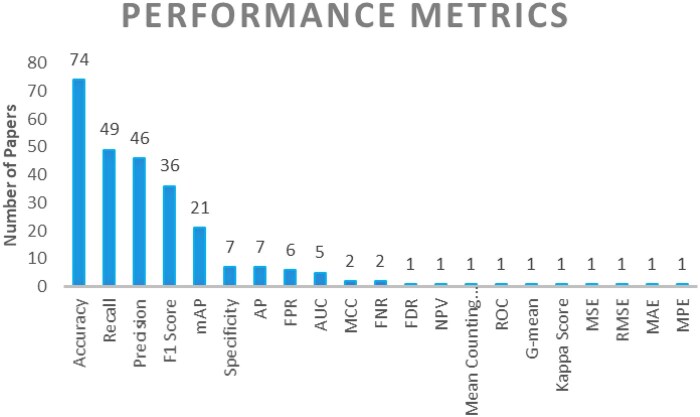
Performance metrics used in the 111 papers.

In the studies that were short-listed, different performance criteria and evaluation methods, as shown in Fig. 8, have been used. Accuracy was the most frequently reported outcome measure in the studies focusing on automatic identification, such as insect classification and plant disease detection. This is not surprising, as accuracy is a basic and widely used metric to assess how well a model performs overall. However, relying solely on accuracy can be misleading, particularly when working with unbalanced datasets where some classes are more common than others (Sokolova and Lapalme 2009).

Other metrics like precision, recall, mean average precision (mAP), and F1-score also appeared regularly, which suggests that some studies adopted more detailed and multiple evaluation methods. These measures are especially relevant when the costs of false positives and false negatives differ such as when identifying harmful versus harmless species. Less commonly reported metrics such as specificity, False Positive Rate, Average Precision, mean counting accuracy, Receiver Operating Characteristic, Area Under Curve, Kappa Score, Mean Squared Error, Root Mean Squared Error, Mean Absolute Error, Mean Percentage Error, Geometric mean, False Negative Rate, False Discovery Rate, Negative Predictive Value, and Matthews Correlation Coefficient indicate that only a portion of the literature considered more comprehensive performance assessments, which are often necessary in health and environmental research (Powers 2011).

## Trends and Insights in AI-Based Insect Classification

The field of automated insect identification is evolving rapidly due to advances in DL and mobile computing. CNNs were used in most image-based systems. Moreover, hybrid architectures are increasingly being investigated for fine-grained classification and improved generalization across species.

It was also observed that hand-crafted features are still used and provide very competitive results in terms of accuracy. Nevertheless, given that classic deep architectures are now easily available, it is not surprising that over 80% of the papers considered used DNN approaches. The architectures vary slightly according to the task addressed. There are still challenges related to the specificity of the databases, the collection of the data, and others related to in natura experiments. Generative AI is also gaining attention as an alternative data augmentation strategy. Overall, the findings regarding performance metrics reflect a gradual shift toward using multiple evaluation criteria in AI-based identification tasks. Encouraging the use of a broader set of performance measures can lead to better validation and more meaningful comparisons across studies.

## Limitations of the Review

The structured review presented in this paper must be interpreted in light of its limitations. Although a rigorous and reproducible approach was applied, the search relied exclusively on the WoS database. Other databases such as Scopus, ScienceDirect, IEEE Xplore, or the ACM Digital Library might have yielded additional relevant studies. Another limitation of this review is that the search strategy relied primarily on the terms “Insect” OR “Insecta,” which may have excluded some relevant studies that use taxon-specific or common names (eg mosquito or pest species). Nevertheless, this approach was adopted to maintain broad taxonomic coverage. In addition, another limitation of this review is that it primarily considers adult insect stages; immature stages were not systematically included because their identification typically requires specialized microscopic imaging and sampling across diverse environmental compartments. Finally, the review was restricted to English-language publications, potentially excluding valuable regional studies, particularly from biodiversity-rich countries where local research is often reported in other languages.

Although multi-author screening and cross-validation were applied to reduce bias, some subjectivity in inclusion decisions is unavoidable. Additionally, the heterogeneity of methodologies, ranging from imaging conditions and dataset structures to evaluation metrics, limits direct performance comparisons between studies. The exclusion of gray literature may also have introduced publication bias, as studies reporting negative or inconclusive results are less likely to appear in indexed databases.

Despite these limitations, the 111 studies included provide a representative overview of the current research landscape. Findings highlight the need for future work addressing a broader taxonomic and geographic scope, improved open and standardized datasets, and deeper investigation into emerging AI paradigms, including generative and explainable models. The structured review presented in this paper must be viewed by considering its limitations.

## Future Research Trends

A brief survey of literature published between September 2024 and August 2025, after completion of this review’s data extraction, indicates that computer vision for insect identification remains an exceptionally active research area. Over 90 papers were published over the year that match the scope of this paper.

The YOLO architecture continues to dominate as the preferred detection framework, with at least 8 studies explicitly referencing it in their titles, confirming its strong position in the field. An emerging trend is the use of images from UAVs. Only 1 paper (Stojnić et al. 2021) in our structured review used such data. Over the past year, other unrelated teams have proposed detection methods tested on UAV imaging (Atanasov et al. 2024, Amarasingam et al. 2025), using strategies that are quite complementary to classic approaches employing YOLO for the same kind of pest insects (Liu et al. 2022). Even in cases where methods were developed and tested on standard RGB images, the future use of UAVs to monitor fields is often cited as a motivation (Berger et al. 2024, Khujamatov et al. 2025).

This trend aligns with the broader observation that more than half of recent studies continue to focus on pest identification, while only a limited subset explicitly address biodiversity monitoring, highlighting a persistent application bias in AI-based entomology.

## Conclusions

The aim of this study was to systematically synthesize computer-vision-based techniques for identifying/classifying adult insecta. The main elements analyzed included biodiversity and taxonomic coverage, the experimental setups, types of optical devices, insect image datasets, and the methodologies for identification/classification. In total, 111 studies were critically analyzed, which corresponded to the period 2012 to 2024.

**RQ1** showed that across the reviewed studies, 122 insect families were represented, spanning 12 orders (Coleoptera, Dermaptera, Diptera, Hemiptera, Hymenoptera, Blattodea (Formerly Isoptera), Lepidoptera, Mantodea, Neuroptera, Odonata, Orthoptera, and Thysanoptera) and 313 genuses with the most frequently studied order being Diptera, Coleoptera, Hemiptera, Hymenoptera and families, including Aphididae, Chrysomelidae, Culicidae, Curculonidae, Delphacidae, Noctuidae (butterfly), Pentatomidae, Tephritidae, Tenebrionidae, using techniques such as macro imaging, wing venation and WIP analysis, or behavioral recognition systems. AI-based insect classification spanned a wide taxonomic spectrum, with applications in pest detection, pollinator monitoring, and vector surveillance.

While answering **RQ2**, the study revealed that the data were collected in either laboratory-based environments or in the field while in some cases, a hybrid setup was used. As a result, several types of traps were used including smart/image-based traps, sticky traps, pheromone traps, light traps, and simulated/visual traps. In terms of optical devices, professional cameras, microscopes, mobile phones, web cams, and even some less conventional image capture devices such as IoT-based cameras, GoPro cameras, scanners, X-ray cameras, CCTV cameras, and dedicated image-acquisition devices developed by the research centers were used in the studies. Future studies might involve a detailed analysis of how the setups, devices or even the insect order affect the accuracy and robustness of automated insect classification systems.

With respect to **RQ3**, public datasets were scarce and geographically skewed. Many articles created their dataset and a few reused their own data across several studies. In some studies, the datasets were built up from online sources that were complemented with the authors’ own image acquisitions. The shortage of datasets could potentially have an impact on the results of image processing and classifications. Future research in this area could identify how the characteristics of the datasets in terms of size, accessibility and geographic coverage influence the generalizability of the models.

Focusing on **RQ4**, we found that the different approaches used for image processing included low-level image processing, shallow ML approaches, full DL approaches, and a mixture of the latter 2. The shallow ML approaches were often based on hand-crafted features such as SIFT or HOG jointly with classic ML methods. Full DL approaches included YOLO, ResNet/EfficientNet/MobileNet, and CNN features. GANs are an emerging option for data augmentation.

We recommend advancing the field through comprehensive reporting beyond overall accuracy (including precision, recall, F1-score, and mAP), the design of lifecycle-aware and domain-adapted models validated under field conditions, the establishment of open and diverse benchmarks with standardized imaging protocols, and the development of frugal, interpretable architectures suitable for deployment in embedded trapping systems.

## Supplementary Material

ieag050_Supplementary_Data
