# Supplementary material for: A structured literature review of computer vision methods for insect identification
Source: J Insect Sci. 2026 Jul 30;26(4):ieag050. doi: 10.1093/jisesa/ieag050 (PMC13424446; doi:10.1093/jisesa/ieag050)
Supplement: ieag050_Supplementary_Data [file ieag050_supplementary_data.zip › References for Supplementary Material 2.docx]

References

1. [Abdelghani BA, Banitaan S, Maleki M, et al. 2021. Kissing bugs Identification using convolutional neural network. IEEE ACCESS. 9:140539-140548.](https://sciwheel.com/work/bibliography/17383130) <https://doi.org/10.1109/ACCESS.2021.3119587>
2. Agarwal M, Al-Shuwaili T, Nugaliyadde A, et al. 2020. Identification and diagnosis of whole body and fragments of *Trogoderma granarium* and *Trogoderma variabile* using visible near infrared hyperspectral imaging technique coupled with deep learning. Comput. Electron. Agric. 173:105438. <https://doi.org/10.1016/j.compag.2020.105438>.
3. [Almryad AS, Kutucu H. 2020. Automatic identification for field butterflies by convolutional neural networks. Eng. Sci. Technol. Int. 23:189**−**195.](https://sciwheel.com/work/bibliography/17365886) <https://doi.org/10.1016/j.jestch.2020.01.006>.
4. [Amrani A, Sohel F, Diepeveen D, et al. 2023. Deep learning-based detection of aphid colonies on plants from a reconstructed *Brassica* image dataset. Comput. Electron. Agric. 205:107587.](https://sciwheel.com/work/bibliography/17604936) <https://doi.org/10.1016/j.compag.2022.107587>.
5. [Asgari, M, Sadeghzadeh A, Islam MB, et al. 2022. Deep learning-based vector mosquitoes classification for preventing infectious diseases transmission. Image Anal. Stereol.](https://sciwheel.com/work/bibliography/16670123) 41:203−215. <https://doi.org/10.5566/ias.2804>.
6. [Badgujar CM, Armstrong PR, Gerken AR, et al. 2023a. Identifying common stored product insects using automated deep learning methods. J. Stored Prod. Res. 103:102166.](https://sciwheel.com/work/bibliography/16989898) <https://doi.org/10.1016/j.jspr.2023.102166>.
7. [Badgujar CM, Armstrong PR, Gerken AR, et al. 2023b. Real-time stored product insect detection and identification using deep learning: System integration and extensibility to mobile platforms. J. Stored Prod. Res. 104:102196.](https://sciwheel.com/work/bibliography/16998214) <https://doi.org/10.1016/j.jspr.2023.102196>
8. [Barboza da Silva C, Naves Silva AA, Barroso G et al. 2021. Convolutional neural networks using enhanced radiographs for real-time detection of *Sitophilus Zeamais* in maize grain. Foods. 10:979.](https://sciwheel.com/work/bibliography/17268204) <https://doi.org/10.3390/foods10040879>
9. [Bisgin H, Bera T, Wu L, et al. 2022. Accurate species identification of food-contaminating beetles with quality-improved elytral images and deep learning. Front. Artif. Intell. 5:952424.](https://sciwheel.com/work/bibliography/17185598) <https://doi.org/10.3389/frai.2022.952424>
10. Bjerge K, Mann HM, Høye TT. 2022. Real‐time insect tracking and monitoring with computer vision and deep learning. Remote Sens. Ecol. Conserv. 8:315−327. <https://doi.org/10.1002/rse2.245>
11. [Bjerge K, Nielsen JB, Sepstrup MV, et al. 2021. An automated light trap to monitor moths (*Lepidoptera*) using computer vision-based tracking and deep learning. Sensors. 21](https://sciwheel.com/work/bibliography/14087899):343 <https://doi.org/10.3390/s21020343>
12. [Blair J, Weiser MD, Kaspari M, et al. 2020. Robust and simplified machine learning identification of pitfall trap-collected ground beetles at the continental scale. Ecol. Evol. 10:13143–13153.](https://sciwheel.com/work/bibliography/17012409) <https://doi.org/10.1002/ece3.6905>
13. Brandt D, Tschaikner M, Chiaburu T, et al. 2024. Low cost machine vision for insect classification. Intelligent Systems Conference; 7−8 June 2023; Amsterdam. Cham: Springer Nature Switzerland. <https://doi.org/10.1007/978-3-031-47715-7_2>
14. Buschbacher K, Ahrens D, Espeland M, et al. 2020. Image-based species identification of wild bees using convolutional neural networks. Ecol. Inform. 55:101017. <https://doi.org/10.1016/j.ecoinf.2019.101017>
15. [Cannet A, Simon Chane C, Akhoundi M, et al. 2022. Wing Interferential Patterns (WIPs) and machine learning, a step toward automatized tsetse (*Glossina* spp.) identification. Sci. Rep. 12: 20086.](https://sciwheel.com/work/bibliography/17555120) <https://doi.org/10.1038/s41598-022-24522-w>
16. [Cannet A, Simon Chane C, Histace A, et al. 2023a. Species identification of phlebotomine sandflies using deep learning and wing interferential pattern (WIP). Sci. Rep. 13:21389.](https://sciwheel.com/work/bibliography/17012423) <https://doi.org/10.1038/s41598-023-48685-2>
17. [Cannet A, Simon Chane C, Histace A, et al. 2023b. Wing Interferential Patterns (WIPs) and machine learning for the classification of some *Aedes* species of medical interest. Sci. Rep. 13:17628.](https://sciwheel.com/work/bibliography/16992831) <https://doi.org/10.1038/s41598-023-44945-3>
18. Cao X, Wei Z, Gao Y, et al. 2020. Recognition of common insect in field based on deep learning. International Conference on Computer Information Science and Application Technology; 17-19 July 2020; Dali, China. IOP Publishing. <https://doi.org/10.1088/1742-6596/1634/1/012034>
19. [Carvajal J A, Romero DG, Sappa AD. 2017. Fine-tuning based deep convolutional networks for Lepidopterous Genus recognition. Progress in Pattern Recognition, Image Analysis, Computer Vision, and Applications; 8−11 November 2016; Lima, Peru. Springer International Publishing, Cham.](https://sciwheel.com/work/bibliography/17269160) <https://doi.org/10.1007/978-3-319-52277-7_57>
20. [Chen JW, Lin WJ, Cheng HJ, et al. 2021. A smartphone-based application for scale pest detection using multiple-object detection methods. Electronics. 10:372.](https://sciwheel.com/work/bibliography/17268187) <https://doi.org/10.3390/electronics10040372>
21. Chen J, Chen W, Nanehkaran, YA, et al. 2024. MAM-IncNet: an end-to-end deep learning detector for Camellia pest recognition. Multimed. Tools Appl. 83:31379-31394. <https://doi.org/10.1007/s11042-023-16680-4>
22. Chulu F, Phiri J, Nkunika PO, et al. 2019. A convolutional neural network for automatic identification and classification of fall army worm moth. Int. J. Adv. Comput. Sci. Appl. 10:112-118. [https://doi.org/10.14569/IJACSA.2019.0100717](https://doi.org/10.1016/j.ecoinf.2022.101587)
23. [Cochero J, Pattori L, Balsalobre A, et al. 2022. A convolutional neural network to recognize Chagas disease vectors using mobile phone images. Ecol. Inform. 68:101587.](https://sciwheel.com/work/bibliography/17268192) <https://doi.org/10.1016/j.ecoinf.2022.101587>
24. Crnojević V, Panić M, Brkljač B, et al. 2014. Image processing method for automatic discrimination of hoverfly species. Math. Probl. Eng. 986271. <https://doi.org/10.1155/2014/986271>
25. Dai F, Wang F, Yang D, et al. 2022. Detection method of citrus Psyllids with field high-definition camera based on improved cascade region-based convolution neural networks. Front. Plant Sci. 12: 816272.<https://doi.org/10.3389/fpls.2021.816272>
26. Dai Q, Cheng X, Qiao Y, et al. 2020. Agricultural pest super-resolution and identification with attention enhanced residual and dense fusion generative and adversarial network. IEEE Access. 8:81943–81959. <https://doi.org/10.1109/ACCESS.2020.2991552>
27. de Geus AR, Batista MA, Rabelo MN, et al. 2019. Maize insects classification through endoscopic video analysis. Advances in Artificial Intelligence: 251-262; 28−31 May, 2019; Kingston, ON, Canada. Springer International Publishing, Cham. <https://doi.org/10.1007/978-3-030-18305-9_20>
28. Ebrahimi MA, Khoshtaghaza MH, Minaei S, et al. 2017. Vision-based pest detection based on SVM classification method. Comput. Electron. Agric. 137:52−58. <https://doi.org/10.1016/j.compag.2017.03.016>
29. Fujisawa T, Noguerales V, Meramveliotakis E, et al. 2023. Image-based taxonomic classification of bulk insect biodiversity samples using deep learning and domain adaptation. Syst. Entomol. 48:387−401. <https://doi.org/10.1111/syen.12583>
30. Gerovichev A, Sadeh A, Winter V, et al. 2021. High throughput data acquisition and deep learning for insect ecoinformatics. Front. Ecol. Evol. 9: 600931. <https://doi.org/10.3389/fevo.2021.600931>
31. Gomes JC, de AB Borges L, Borges DL, et al. 2023. A multi-layer feature fusion method for few-shot image classification. Sensors. 23:6880. <https://doi.org/10.3390/s23156880>
32. Gong H, Liu T, Luo T, et al. 2023. Based on FCN and DenseNet framework for the research of rice pest identification methods. Agronomy. 13:410. <https://doi.org/10.3390/agronomy13020410>
33. Guerron A, Benitez DS, Zapata S, et al. 2016. Image processing algorithm for improving the identification of patterns on Diptera wings. International Autumn Meeting on Power, Electronics and Computing: 1-6; 09−11 November 2016; Ixtapa, Mexico. IEEE. <https://doi.org/10.1109/ROPEC.2016.7830519>
34. Gupta YM, Homchan S,. 2021. Short communication: Insect detection using a machine learning model. Nus. Biosci. 13: 68-72. <https://doi.org/10.13057/nusbiosci/n130110>.
35. Hadi MK, Kassim MSM, Wayayok A, 2021. Development of an automated multidirectional pest sampling detection system using motorized sticky traps. IEEE Access. 9:67391−67404. <https://doi.org/10.1109/ACCESS.2021.3074083>
36. Hansen MF, Oparaeke A, Gallagher R, et al. 2022. Towards machine vision for insect welfare monitoring and behavioural insights. Front. Vet. Sci. 9:835529 <https://doi.org/10.3389/fvets.2022.835529>
37. He Y, Zhou Z, Tian L, et al. 2020. Brown rice planthopper (*Nilaparvata lugens* Stal) detection based on deep learning. Precis. Agric. 21(6):1385-1402. <https://doi.org/10.1007/s11119-020-09726-2>
38. Hong SJ, Nam I, Kim SY, et al. 2021. Automatic pest counting from pheromone trap images using deep learning object detectors for *Matsucoccus Thunbergianae* monitoring. Insects. 12(4): 342. [https://doi.org/10.3390/insects12040342](https://doi.org/10.3390/insects12040).
39. Huang JH, Liu YT, Ni HC, et al. 2021. Termite pest identification method based on deep convolution neural networks. J. Econ. Entomol. 114: 2452–2459. <https://doi.org/10.1093/jee/toab162>
40. Huang ML, Chuang TC, Liao YC. 2022. Application of transfer learning and image augmentation technology for tomato pest identification. Sustain. Comput.: Inform. Syst. 33: 100646. <https://doi.org/10.1016/j.suscom.2021.100646>.
41. Huddar SR, Gowri S, Keerthana K, et al. 2012. Novel algorithm for segmentation and automatic identification of pests on plants using image processing. Third International Conference on Computing, Communication and Networking Technologies: 1-5; 26–28 July 2012; Coimbatore, India. IEEE. <https://doi.org/10.1109/ICCCNT.2012.6396012>.
42. Ikeda M, Ruedeeniraman N, Barolli L. 2021. An intelligent VegeCareAI tool for next generation plant growth management. Internet Things. 14:100381. <https://doi.org/10.1016/j.iot.2021.100381>.
43. Isawasan P, Abdullah ZI, Ong SQ, et al. 2023. A protocol for developing a classification system of mosquitoes using transfer learning. MethodsX. 10: 101947. <https://doi.org/10.1016/j.mex.2022.101947>
44. Jin X, Zhu X, Ji J, et al. 2024. Online diagnosis platform for tomato seedling diseases in greenhouse production. Int. J. Agric. Biol. Eng. 17(1): 80-89. <https://doi.org/10.25165/j.ijabe.20241701.8433>.
45. Kalfas I, De Ketelaere B, Bunkens K, et al. 2023. Towards automatic insect monitoring on witloof chicory fields using sticky plate image analysis. Ecol. Inform. 75: 102037. <https://doi.org/10.1016/j.ecoinf.2023.102037>.
46. Kargar A, Zorbas D, Tedesco S, et al. 2024. Detecting *Halyomorpha halys* using a low-power edge-based monitoring system. Comput. Electron. Agric. 221: 108935. <https://doi.org/10.1016/j.compag.2024.108935>.
47. Kathole AB, Vhatkar KN, Patil SD. 2024. IoT-enabled pest identification and classification with new meta-heuristic-based deep learning framework. Cybern. Syst. 55: 380–408. <https://doi.org/10.1080/01969722.2022.2122001>.
48. Lee S, Kim H, Cho BK. 2023. Deep learning-based image classification for major mosquito species inhabiting Korea. Insects. 14(6): 526. <https://doi.org/10.3390/insects140>.
49. Li H, Liang Y, Liu Y, et al. 2023. Development of an intelligent field investigation system for *Liriomyza* using SeResNet-Liriomyza for accurate identification. Comput. Electron. Agric. 214: 108276. <https://doi.org/10.1016/j.compag.2023.108276>.
50. Li R, Wang R, Zhang J, et al. 2019. An effective data augmentation strategy for CNN-based pest localization and recognition in the field. IEEE Access. 7: 160274-160283. <https://doi.org/10.1109/ACCESS.2019.2949852>.
51. Li W, Wang D, Li M, et al. 2021. Field detection of tiny pests from sticky trap images using deep learning in agricultural greenhouse. Comput. Electron. Agric. 183: 106048. <https://doi.org/10.1016/j.compag.2021.106048>.
52. Li W, Yang Z, Lv J, et al. 2022. Detection of small-sized insects in sticky trapping images using spectral residual model and machine learning. Front. Plant Sci. 13: 915543. <https://doi.org/10.3389/fpls.2022.915543>.
53. Lim S, Kim S, Park S, et al. 2018. Development of application for forest insect classification using CNN. 15th International Conference on Control, Automation, Robotics and Vision (ICARCV 2018): 1128-1131; 18–21 Nov 2018; Singapore. IEEE. <https://doi.org/10.1109/ICARCV.2018.8581103>
54. Linfeng W, Yong L, Jiayao L, et al. 2023. Based on the multi-scale information sharing network of fine-grained attention for agricultural pest detection. PLoS ONE. 18: e0286732. 23. <https://doi.org/10.1371/journal.pone.0286732>.
55. Liu C, Zhai Z, Zhang R, et al. 2022a. Field pest monitoring and forecasting system for pest control. Front. Plant Sci. 13: 990965. [https://doi.org/10.3389/fpls.2022.990965.](https://doi.org/10.3389/fpls.2022.990965)
56. Liu H, Chahl JS. 2018. A multispectral machine vision system for invertebrate detection on green leaves. Comput. Electron. Agric. 150: 279-288. <https://doi.org/10.1016/j.compag.2018.05.002>.
57. Liu J, Wang X, Miao W, et al. 2022b. Tomato pest recognition algorithm based on improved YOLOv4. Front. Plant Sci. 13: 814681. <https://doi.org/10.3389/fpls.2022.814681>.
58. Liu L, Wang R, Xie C, et al. 2019. PestNet: an end-to-end deep learning approach for large-scale multi-class pest detection and classification. IEEE Access. 7: 45301–45312. [https://doi.org/10.1109/ACCESS.2019.2909522](http://doi.org/10.1109/ACCESS.2019.2909522)
59. Lu CY, Rustia DJA, Lin TT. 2019. Generative adversarial network based image augmentation for insect pest classification enhancement. IFAC-PapersOnLine. 52(30): 1-5. [https://doi.org/10.1016/j.ifacol.2019.12.406](http://doi.org/10.1016/j.ifacol.2019.12.406).
60. Machraoui AN, Diouani MF, Mouelhi A, et al. 2019. Automatic identification and behavioral analysis of phlebotomine sand flies using trajectory features. Vis. Comput. 35(5): 721-738. <https://doi.org/10.1007/s00371-018-1506-x>.
61. Mamdouh N, Khattab A. 2021. YOLO-based deep learning framework for olive fruit fly detection and counting. IEEE Access. 9: 84252–84262. <https://doi.org/10.1109/ACCESS.2021.3088075>.
62. Martins VA, Freitas LC, de Aguiar MS, et al. 2019. Deep learning applied to the identification of fruit fly in intelligent traps. 2019 IX Brazilian symposium on computing systems engineering (SBESC); 19-22 Nov. 2019; Brazil. IEEE. <https://doi.org/10.1109/SBESC49506.2019.9046088>
63. Mottos AB, Feris RS. 2014. Fusing well-crafted feature descriptors for efficient fine-grained classification. 2014 IEEE International Conference on Image Processing (ICIP); 27-30 Oct. 2014; Paris, France. IEEE. <https://doi.org/10.1109/ICIP.2014.7026052>.
64. Murali N, Schneider J, Levine J, et al. 2019. Classification and re-identification of fruit fly individuals across days with convolutional neural networks. The 2019 IEEE Winter Conference on Applications of Computer Vision (WACV); 7-11 Jan. 2019; Waikoloa Village, USA. IEEE. <https://doi.org/10.1109/WACV.2019.00066>.
65. Nasir A, Ullah MO, Yousaf MH. 2023. AI in apiculture: A novel framework for recognition of invasive insects under unconstrained flying conditions for smart beehives. Eng. Appl. Artif. Intell. 119: 105784. [https://doi.org/10.1016/j.engappai.2022.105784.](https://doi.org/10.1016/j.engappai.2022.105784)
66. Nazri A, Mazlan N, Muharam F. 2018. PENYEK: Automated brown planthopper detection from imperfect sticky pad images using deep convolutional neural network. PLoS ONE. 13: e0208501. <https://doi.org/10.1371/journal.pone.0208501>.
67. Niyigena G, Lee S, Kwon S, et al. 2023. Real-time detection and classification of *Scirtothrips Dorsalis* on fruit crops with smartphone-based deep learning system: Preliminary Results. Insects. 14:523. <https://doi.org/10.3390/insects14060523>.
68. Ong SQ, Ahmad H. 2022. An annotated image dataset of medically and forensically important flies for deep learning model training. Sci. Data. 9: 510. <https://doi.org/10.1038/s41597-022-01627-5>.
69. Ong SQ, Hamid SA. 2022. Next generation insect taxonomic classification by comparing different deep learning algorithms. PLoS ONE. 17: e0279094. <https://doi.org/10.1371/journal.pone.0279094>.
70. Park J, Kim DI, Choi B et al. 2020. Classification and morphological analysis of vector mosquitoes using deep convolutional neural networks. Sci. Rep. 10: 1012. <https://doi.org/10.1038/s41598-020-57875-1>.
71. Park YH, Choi SH, Kwon YJ, et al. 2023. Detection of soybean insect pest and a forecasting platform using deep learning with unmanned ground vehicles. Agronomy. 13: 477. <https://doi.org/10.3390/agronomy13020477>.
72. Pise R, Patil K. 2023. A deep transfer learning framework for the multi-class classification of vector mosquito species. J. Ecol. Eng. 24: 183–191. [https://doi.org/10.12911/22998993/168501.](https://doi.org/10.12911/22998993/168501)
73. Popkov A, Konstantinov F, Neimorovets V, et al. 2022. Machine learning for expert-level image-based identification of very similar species in the hyperdiverse plant bug family Miridae (Hemiptera: Heteroptera). Syst. Entomol. 47: 487–503. <https://doi.org/10.1111/syen.12543>.
74. Preti M, Moretti C, Scarton G, et al. 2021. Developing a smart trap prototype equipped with camera for tortricid pests remote monitoring. Bull. Insectology. 74: 147–160
75. Qing Y, Feng J, Tang J, et al. 2020. Development of an automatic monitoring system for rice light-trap pests based on machine vision. J. Integr. Agric. 19(10): 2500–2513. [https://doi.org/10.1016/S2095-3119(20)63168-9.](https://doi.org/10.1016/S2095-3119(20)63168-9)
76. Ramalingam B, Mohan RE, Pookkuttath S, et al. 2020. Remote insects trap monitoring system using deep learning framework and IoT. Sensors. 20(18): 5280. <https://doi.org/10.3390/s20185280>.
77. Ratnayake MN, Dyer AG, Dorin A 2021. Tracking individual honeybees among wildflower clusters with computer vision-facilitated pollinator monitoring. PLoS ONE. 16: e0239504. <https://doi.org/10.1371/journal.pone.0239504>.
78. Remboski TB, de Souza WD, de Aguiar MS, et al. 2018. Identification of fruit fly in intelligent traps using techniques of digital image processing and machine learning. The 33rd Annual ACM Symposium on Applied Computing; 2018; Pau, France. ACM. [https://doi.org/10.1145/3167132.3167155.](https://doi.org/10.1145/3167132.3167155)
79. Rimal K, Shah KB, Jha AK 2023. Advanced multi-class deep learning convolution neural network approach for insect pest classification using TensorFlow. Int. J. Environ. Sci. Technol. 20: 4003–4016. <https://doi.org/10.1007/s13762-022-04277-7>.
80. Rodríguez LAR, Castañeda-Miranda CL, Lució MM, et al. 2021. Quarternion color image processing as an alternative to classical grayscale conversion approaches for pest detection using yellow sticky traps. Math. Comput. Simul. 182: 646–660. <https://doi.org/10.1016/j.matcom.2020.11.022>.
81. Rossi de Gasperis S, Carpaneto GM, Nigro G, et al. 2017. Computer-aided photographic identification of *Rosalia Alpina* (Coleoptera: Cerambycidae) applied to a mark-recapture study. Insect Conserv. Divers. 10(1): 54–63. <https://doi.org/10.1111/icad.12199>.
82. Rustia DJA, Chao JJ, Chiu LY, et al. 2020. Automatic greenhouse insect pest detection and recognition based on a cascaded deep learning classification method. J. Appl. Entomol. 145(3): 206–222. <https://doi.org/10.1111/jen.12834>
83. Rustia DJA, Lee WC, Lu CY, et al. 2023. Edge-based wireless imaging system for continuous monitoring of insect pests in a remote outdoor mango orchard. Comput. Electron. Agric. 211: 108019. <https://doi.org/10.1016/j.compag.2023.108019>
84. Shen Y, Hossain MZ, Ahmed KA, et al. 2024. An open set model for pest identification. Comput. Biol. Chem. 108: 108002. <https://doi.org/10.1016/j.compbiolchem.2023.108002>
85. Shen Y, Zhou H, Li J, et al. 2018. Detection of stored-grain insects using deep learning. Comput. Electron. Agric. 145: 319–325. <https://doi.org/10.1109/ACCESS.2020.3021830>
86. Shirali H, Hübner J, Both R, et al. 2024. Image-based recognition of parasitoid wasps using advanced neural networks. Invertebr. Syst. 38(6): IS24011.<https://doi.org/10.1071/IS24011>
87. Silva A, Meireles S, Silva S 2020. Using partial least squares in butterfly species identification. In 2020 33rd SIBGRAPI Conference on Graphics, Patterns and Images (SIBGRAPI). 7–10 Nov 2020. IEEE. <https://doi.org/10.1109/AST66626.2025.00023>
88. Sittinger M, Uhler J, Pink M, et al. 2024. Insect Detect: an open-source DIY camera trap for automated insect monitoring. PLoS ONE. 19(4): e0295474. <https://doi.org/10.1371/journal.pone.0295474>
89. Stojnić V, Risojević V, Muštra M, et al. 2021. A method for detection of small moving objects in UAV videos. Remote Sens. (Basel). 13: 653.<https://doi.org/10.3390/rs13040653>
90. Sun G, Liu S, Luo H, et al. 2022. Intelligent monitoring system of migratory pests based on searchlight trap and machine vision. Front. Plant Sci. 13: 897739. <https://doi.org/10.3389/fpls.2022.897739>
91. Sun Y, Liu X, Yuan M, et al. 2018. Automatic in-trap pest detection using deep learning for pheromone-based *Dendroctonus valens* monitoring. Biosyst. Eng. 176: 140–150. <https://doi.org/10.1016/j.biosystemseng.2018.10.012>
92. Tan S, Hu S, He S, et al. 2024. Leveraging hyperspectral images for accurate insect classification with a novel two-branch self-correlation approach. Agronomy. 14: 863. <https://doi.org/10.3390/agronomy14040863>
93. Tannous M, Stefanini C, Romano D. 2023. A deep-learning-based detection approach for the identification of insect species of economic importance. Insects. 14: 148. <https://doi.org/10.3390/insects14020148>
94. Truong QB, Thanh TKN, Nguyen MT, et al. 2018. Shallow and deep learning architecture for pests identification on pomelo leaf. 10th International Conference on Knowledge and Systems Engineering (KSE 2018); 1–3 November 2018; Ho Chi Minh City, Vietnam. IEEE. <https://doi.org/10.1109/KSE.2018.8573422>
95. Tuda M, Luna-Maldonado AI. 2020. Image-based insect species and gender classification by trained supervised machine learning algorithms. Ecol. Inform. 60: 101135. <https://doi.org/10.1016/j.ecoinf.2020.101135>
96. Venegas P, Calderon F, Riofrío D, et al. 2021. Automatic ladybird beetle detection using deep-learning models. PLoS ONE. 16: e0253027. <https://doi.org/10.1371/journal.pone.0253027>
97. Wang J, Chen Y, Hou X, et al. 2021. An intelligent identification system combining image and DNA sequence methods for fruit flies with economic importance (Diptera: Tephritidae). Pest Manag. Sci. 77(7): 3382–3395. <https://doi.org/10.1002/ps.6383>
98. Wang J, Kang R, Chen K, et al. 2017. Automatic identification of Asian rice plant-hopper based on image processing. Appl. Eng. Agric. 33: 591–602. <http://doi.org/10.13031/aea.11605>
99. Wang J, Li Y, Feng H, et al. 2020. Common pests image recognition based on deep convolutional neural network. Comput. Electron. Agric. 179: 105834. <https://doi.org/10.1016/j.compag.2020.105834>
100. Wang T, Zhao L, Li B, et al. 2022. Recognition and counting of typical apple pests based on deep learning. Ecol. Inform. 68: 101556. <https://doi.org/10.1016/j.ecoinf.2022.101556>
101. Wang X, Ma Z, Xing Y, et al. 2024. Rapid species discrimination of similar insects using hyperspectral imaging and lightweight edge artificial intelligence. R. Soc. Open Sci. 11: 240485. <https://doi.org/10.1098/rsos.240485>
102. Wen C, Guyer D. 2012. Image-based orchard insect automated identification and classification method. Comput. Electron. Agric. 89: 110–115. <https://doi.org/10.1016/j.compag.2012.08.008>
103. Wu X, Zhan C, Lai YK, et al. 2019. IP102: A large-scale benchmark dataset for insect pest recognition. 2019 IEEE/CVF Conference on Computer Vision and Pattern Recognition (CVPR); 15–20 June 2019; Long Beach CA USA. IEEE. <https://doi.org/10.1109/CVPR.2019.00899>
104. Xiong H, Li J, Wang T, et al. 2024. EResNet-SVM: an overfitting-relieved deep learning model for recognition of plant diseases and pests. J. Sci. Food Agric. 104: 6018–6034. <https://doi.org/10.1002/jsfa.13462>
105. Yalcin H. 2015. Vision based automatic inspection of insects in pheromone traps. Fourth International Conference on Agro-Geoinformatics; 20–24 July 2015; Istanbul, Turkey. IEEE. <https://doi.org/10.1109/Agro-Geoinformatics.2015.7248113>
106. Yang H, Li Y, Xin L, et al. 2023. Mcsnet+: enhanced convolutional neural network for detection and classification of *Tribolium* and *Sitophilus* sibling species in actual wheat storage environments. Foods. 12: 3653.<https://doi.org/10.3390/foods12193653>
107. Yu J, Shen Y, Liu N, et al. 2022. Frequency-enhanced channel-spatial attention module for grain pests classification. Agriculture. 12: 2046. <https://doi.org/10.3390/agriculture12122046>
108. Zhang H, Zhao S, Song Y, et al. 2022. A deep learning and Grad-Cam-based approach for accurate identification of the fall armyworm (*Spodoptera frugiperda*) in maize fields. Comput. Electron. Agric. 202: 107440. <https://doi.org/10.1016/j.compag.2022.107440>
109. Zhang X, Li Z, Ren L, et al. 2024. Detection and recognition of the invasive species *Hylurgus ligniperda* in traps based on a cascaded convolution neural network. Pest Manag. Sci. 80: 4223–4230. <https://doi.org/10.1002/ps.8126>
110. Zhu LQ, Ma MY, Zhang Z, et al. 2013. Using CART and LLC for image recognition of Lepidoptera. Pan-Pac Entomol. 89(3): 176–186. <https://doi.org/10.3956/2013-08.1>
111. Zhu LQ, Ma MY, Zhang Z, et al. 2016. Hybrid deep learning for automated lepidopteran insect image classification. Orient Insects. 51: 79–91. <https://doi.org/10.1080/00305316.2016.1252805>
