# Supplementary material for: A structured literature review of computer vision methods for insect identification
Source: J Insect Sci. 2026 Jul 30;26(4):ieag050. doi: 10.1093/jisesa/ieag050 (PMC13424446; doi:10.1093/jisesa/ieag050)
Supplement: ieag050_Supplementary_Data [file ieag050_supplementary_data.zip › Supplementary Material 1.docx]

Supplementary Material 1

Quality Assessment Criteria

| **Number** | **Quality Criteria** | **Score** | | | **Weightage** |
| --- | --- | --- | --- | --- | --- |
|  |  | **0** | **1** | **2** |  |
| QC1 | Taxonomy/ methodology | No mention of the methodology | Methodology mentioned briefly or only one method mentioned | Methodology described fully with at least two methods mentioned | 1 |
| QC2 | Image capture approach/devices | Not mentioned | Image capture mentioned but with no details about devices | Specifications of devices described in detail | 1 |
| QC3 | Image processing or computer vision technique | Techniques not described | Brief description of techniques | Detailed description of techniques | 2 |
| QC4 | Number of processed samples | Not mentioned | Overall number of samples mentioned but with no sub categorisation | Clear indication on total number of samples used and sub categorisation | 1 |
| QC5 | Performance evaluation | No evaluation performed | Brief description of evaluation methods, without values for performance metrics | Detailed description of evaluation methods, with values for performance metrics | 2 |
